# Three-dimensional printed PLA scaffold and human gingival stem cell-derived extracellular vesicles: a new tool for bone defect repair

**DOI:** 10.1186/s13287-018-0850-0

**Published:** 2018-04-13

**Authors:** Francesca Diomede, Agnese Gugliandolo, Paolo Cardelli, Ilaria Merciaro, Valeria Ettorre, Tonino Traini, Rossella Bedini, Domenico Scionti, Alessia Bramanti, Antonio Nanci, Sergio Caputi, Antonella Fontana, Emanuela Mazzon, Oriana Trubiani

**Affiliations:** 1 Department of Medical, Oral and Biotechnological Sciences, University “G. d’Annunzio”, Chieti, Italy; 2 IRCCS Centro Neurolesi “Bonino Pulejo”, Messina, Italy; 3 Department of Pharmacy, University “G. d’Annunzio”, Chieti, Italy; 4 National Centre of Innovative Technologies in Public Health, Italian National Institute of Health, Rome, Italy; 5 Institute of Applied Science and Intelligent Systems “ISASI Eduardo Caianiello”, CNR, Messina, Italy; 6 Laboratory for the study of Calcified Tissues and Biomaterials, Department of Stomatology, Faculty of Dentistry, Université de Montréal, Montréal, Québec, Canada; 7 Department of Medical, Oral and Biotechnological Sciences, University “G. d’Annunzio”, Via dei Vestini, 66100 Chieti, Italy

**Keywords:** Human gingival mesenchymal stem cells, 3D scaffold, Extracellular vesicles, Bone regeneration

## Abstract

**Background:**

The role of bone tissue engineering in the field of regenerative medicine has been a main research topic over the past few years. There has been much interest in the use of three-dimensional (3D) engineered scaffolds (PLA) complexed with human gingival mesenchymal stem cells (hGMSCs) as a new therapeutic strategy to improve bone tissue regeneration. These devices can mimic a more favorable endogenous microenvironment for cells in vivo by providing 3D substrates which are able to support cell survival, proliferation and differentiation. The present study evaluated the in vitro and in vivo capability of bone defect regeneration of 3D PLA, hGMSCs, extracellular vesicles (EVs), or polyethyleneimine (PEI)-engineered EVs (PEI-EVs) in the following experimental groups: 3D-PLA, 3D-PLA + hGMSCs, 3D-PLA + EVs, 3D-PLA + EVs + hGMSCs, 3D-PLA + PEI-EVs, 3D-PLA + PEI-EVs + hGMSCs.

**Methods:**

The structural parameters of the scaffold were evaluated using both scanning electron microscopy and nondestructive microcomputed tomography. Nanotopographic surface features were investigated by means of atomic force microscopy. Scaffolds showed a statistically significant mass loss along the 112-day evaluation.

**Results:**

Our in vitro results revealed that both 3D-PLA + EVs + hGMSCs and 3D-PLA + PEI-EVs + hGMSCs showed no cytotoxicity. However, 3D-PLA + PEI-EVs + hGMSCs exhibited greater osteogenic inductivity as revealed by morphological evaluation and transcriptomic analysis performed by next-generation sequencing (NGS). In addition, in vivo results showed that 3D-PLA + PEI-EVs + hGMSCs and 3D-PLA + PEI-EVs scaffolds implanted in rats subjected to cortical calvaria bone tissue damage were able to improve bone healing by showing better osteogenic properties. These results were supported also by computed tomography evaluation that revealed the repair of bone calvaria damage.

**Conclusion:**

The re-establishing of the integrity of the bone lesions could be a promising strategy in the treatment of accidental or surgery trauma, especially for cranial bones.

**Electronic supplementary material:**

The online version of this article (10.1186/s13287-018-0850-0) contains supplementary material, which is available to authorized users.

## Background

Bone defects are serious consequences of conditions such as trauma, infection, surgical resections, and other systemic problems that negatively affect the bone healing process [1]. In particular, calvarial bone lesions due to accidental or surgery trauma represent a major and difficult health concern in reconstructive surgery [2–4]. Indeed, it is well known that the spontaneous calvaria regeneration occurs only in children less than 2 years old [2]. Thus, surgeons have been trying for many years to restore functionality and the aesthetic appearance using autografts, allografts, and even xenografts without satisfactory outcomes [2]. Consequently, there has been considerable effort towards developing new strategies to improve bone growth, bone healing, and repair cranial defects. In this context, tissue engineering has become a promising approach for bone regeneration. In particular, the use of scaffolds represents an integral part of bone tissue engineering [5–7]. Scaffolds offer mechanical support and three-dimensional (3D) support that favor cell adhesion, migration, and differentiation in vivo [8, 9]. However, to be used in the regeneration of tissues, these devices must have some fundamental features including biocompatibility, biodegradability, mechanical strength, and matrix properties. Fiber and pore sizes may influence some cellular responses, including migration, proliferation, and differentiation [10, 11]. For these reasons, there is increasing interest in designing new biomaterials that could be used in the form of scaffolds as bone substitutes conceived to induce minimal or no immune response and for encouraging implant/tissue interaction [12]. The most used synthetic and biodegradable scaffolds are poly(ε-caprolactone), poly(glycolic acid) and poly(lactide) (PLA) scaffolds as well as their copolymers [13, 14]. Among these, PLA is widely used in the regenerative medicine field due to its biodegradability, biocompatibility, thermal plasticity, and suitable mechanical properties [15].

Human dental mesenchymal stem cells (MSCs) derived mainly from gingiva and periodontal ligament hold great promise for bone regeneration owing to the less invasive method for tissue explant collection and their capacity to be a simple autologous MSC resource tool [16, 17]. These cells possess a high capacity for expansion and the ability to differentiate into osteogenic cells that can grow on biocompatible substrates [18, 19]; their therapeutic efficacy has been evaluated in regenerative medicine relative to both dental and nondental applications [20].

There are now considerable data supporting the concept of paracrine signaling of extracellular vesicles (EVs) as an important factor for stem cell therapy [21, 22]. EVs are small membrane vesicles containing functional proteins, lipids, and nucleic acids, such as mRNA and microRNA (miRNA), which are released by a variety of cell types [23, 24]. The soluble bioactive molecules present in EVs directly activate target cells, inhibit apoptosis and fibrosis, and stimulate tissue-intrinsic progenitor cell differentiation [25]. Recently, Qin et al. demonstrated that human MSC-derived EVs enter the osteoblasts and deliver osteogenic miRNA by endocytosis, thus modulating osteogenic gene expression. Therefore, human MSCs are recognized as being able to promote bone regeneration in Sprague-Dawley rats with calvarial defects [26] or fracture healing in a mouse model [27], and are potently proangiogenic [28]. Nevertheless, in these studies EVs were either directly injected to the fracture [27], or applied using hydrogel as a delivery system [26]. Only Xie et al. [28] used a proper scaffold (decalcified bone matrix) to properly deliver EVs.

Our aim was to evaluate the regenerative effects of 3D PLA scaffolds enriched with human gingival MSCs (hGMSCs) and complexed with EVs as well as with engineered EVs. In particular, we sought to engineer EVs to improve the adhesiveness of EVs onto the 3D PLA scaffold and to favor the intracellular release of EVs content. We have directed our attention towards the achievement of a coating of polyethyleneimine (PEI), a biocompatible polymer that is known in PEI-complexed nucleic acids to induce osmotic swelling (i.e., a proton-sponge effect) and promote the endosomal content release without the need for an additional endosomolytic agent [29]. Moreover, PEI has been used for activating PLA scaffolds and has been demonstrated to have an affinity for them [30]. Altogether, our in vivo and in vitro analyses show that EVs and PEI-EVs can be advantageously used with PLA scaffolds to promote bone regeneration.

## Methods

### Scaffold development and three-dimensional scaffold constructs

The scaffold was obtained from a commercial poly(lactide) (PLA; Keytech srl, Ancona, Italy). This material was provided as a 1.75-mm diameter filament. Industrial-grade PLA is generally a stereocopolymer with some percentage of d-units in the poly (l-lactide) chain and has higher molar mass distribution. This material is also formulated with additives, such as a stabilizer and a nucleating agent, the latter being important for processing and stability [31]. Technical specifications of the PLA filament are reported in Table 1.

**Table 1:** Physical characteristics of the investigated poly(lactide)

Property	Test standard	Unit	Values (50% RH)
Tensile strength	ISO 527	MPa	52
Elongation strength	ISO 527	%	> 20
Flexural stress	ISO 178	MPa	85
IZOD impact, notched	ISO 180/1A	kJ/m^2^	22
H.D.T. method A (1.8 MPa)	ISO 75	°C	65
Density	ISO 1183	g/cm^3^	1,25
Fire resistance (3.2 mm)	UL 94	–	HB
Melt temperature range	–	°C	220–230

**Fig. 1:**
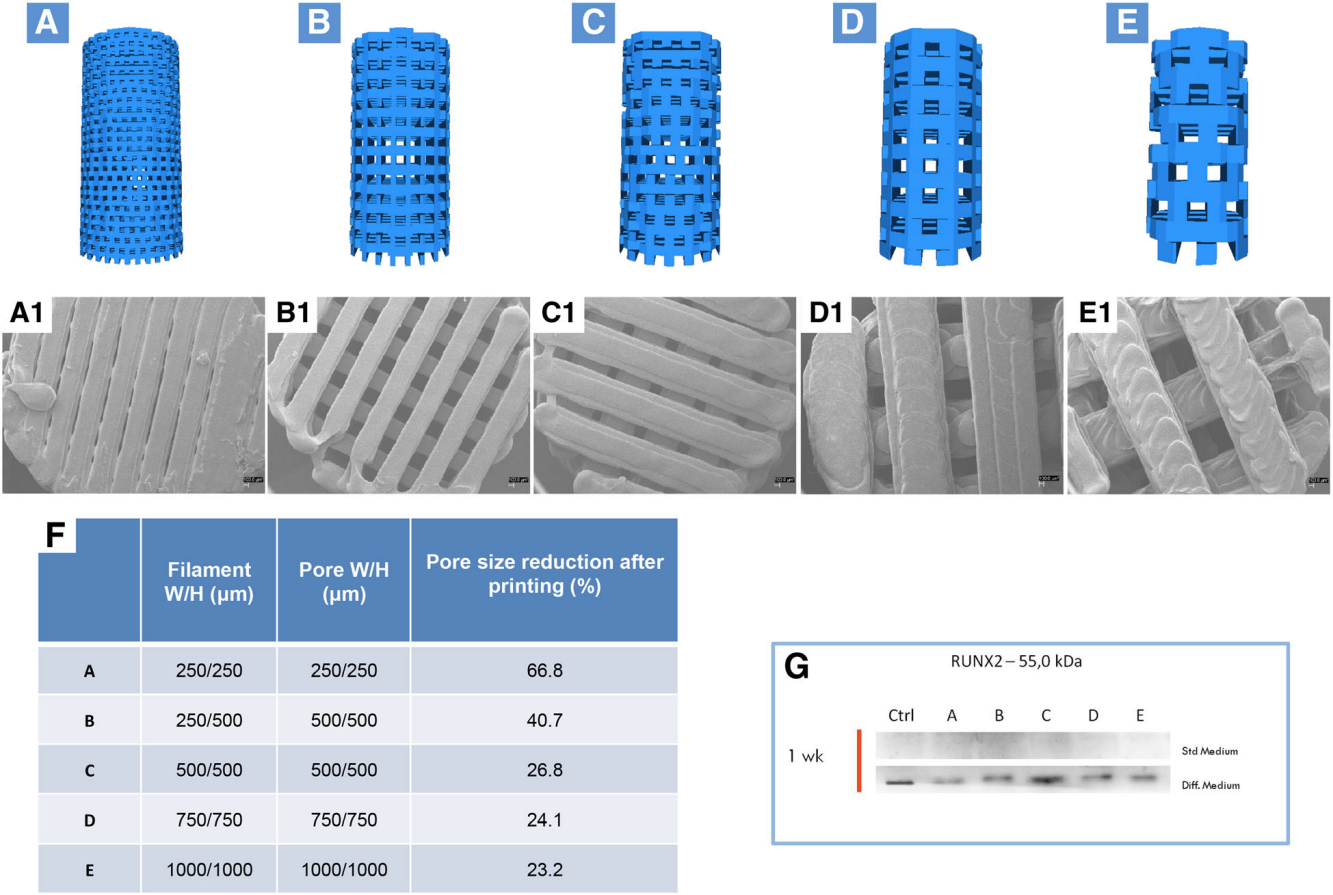
Design of standardized three-dimensional modular scaffolds (3D PLA) with different porosity and filament dimension. **a** Filament width/height 250/250 μm and pore width/height 250/250 μm; **b** Filament width/height 250/500 μm and pore width/height 250/500 μm; **c** Filament width/height 500/500 μm and pore width/height 500/500 μm; **d** Filament width/height 750/750 μm and pore width/height 750/750 μm; **e** Filament width/height 1000/1000 μm and pore width/height 1000/1000 μm. **a1–e1** SEM micrographs of samples from **a–e**. Pore size reduction is present in each group. In-filament layering is observed especially in samples **b–e**. **f** Table with filament, porosity, and pore size reduction specifications. **g** RUNX2 expression in hGMSCs cultured in control and osteogenic medium. An increased expression of RUNX2 was present in cells cultured with scaffold design C (**c**)

Different scaffold designs were investigated in this study. The main differences were 3D structure, pore size, and porosity. Height and diameter of the samples were kept constant at 12 and 6 mm, respectively. The different design characteristics for each sample are reported in Fig. 1f. Each sample was developed with a commercial CAD software (Rhinoceros 5, McNeel Europe, Barcelona, Spain); the projects were then applied to a printing slicing software (Cura 15.04, Ultimaker B.V., Geldermalsen, The Netherlands). The sliced project was finally transferred to a commercial fuse filament fabrication 3D printer (DeltaWASP 2040; CSP srl, Massa Lombarda, Italy).

### Structural scaffold evaluation

#### Scaffold scanning electron microscope characterization

A scanning electron microscope (EVO 50 XVP; Zeiss, Jena, Germany) was used to image the surfaces of the scaffold; the specimens were sputtered with 4–8 nm of gold and then mounted on carbon tape dots [32].

#### In vitro osteogenesis performance

hGMSCs were seeded at 8 × 10^3^ cells/cm^2^ in chemically defined MSC growth medium (MSCGM-CD) (control medium) (Lonza, Basel, Switzerland) and in osteogenic differentiation medium (Lonza) in the presence of all scaffold designs (Fig. 1a–e). To evaluate the performance of different scaffold designs in terms of osteogenic differentiation, the expression of RUNX2 after 1 week of culture was performed by Western blotting analysis (Fig. 1g).

#### In vitro degradation

Scaffolds were immersed into 20 mL of phosphate-buffered saline (PBS; 0.01 M, pH 7.4) and ascorbic acid (0.01 M) to evaluate in vitro degradation as previously reported. The function of the ascorbic acid was to stabilize the scaffold degradation byproducts. Vials were kept at 37 °C on a shaker table at 75 rpm. At each evaluation time point (days 1, 7, 14, 28, 56, 84, and 112), pH was measured, PBS was replaced, and scaffolds were evaluated for residual mass after vacuum drying. To evaluate mechanical and structural characteristics of the degrading scaffolds, wet scaffolds were evaluated in compression (*n* = 5); scaffold mean pore size, trabecular thickness, and porosity were evaluated using microcomputed tomography (mCT) at day 0 (*n* = 3).

#### Compressive load

Scaffolds from each group underwent compressive mechanical testing at each degradation time point using a universal testing machine (LR 30 K; Lloyd Instruments Ltd., Bognor Regis, UK). At each time point, the scaffolds were removed from PBS and immediately tested in air except on day 0 (*n* = 5), when testing was performed before immersion in PBS. For all tests the machine was equipped with a 500 N load cell. Samples were loaded at a crosshead speed of 0.5 mm/min and experimental data were acquired every 10 ms. Yield strength and compressive modulus were evaluated. Moreover, strength at 15% strain was measured since no bending of the specimens was observed until this strain value.

#### Microcomputed tomography

mCT was used to obtain noninvasive images of the scaffolds. mCT analysis was performed on a Skyscan 1072 (Bruker MicroCT, Kontich, Belgium) operated at 70 kV/200 mA, 0.45° rotation step, with a total rotation angle of 180°.

### Cell culture and derived extracellular vesicles

Written approval for gingival biopsy collection was obtained from the Medical Ethics Committee at the Medical School, “G. d’Annunzio” University, Italy, and each participant gave informed consent. Gingival tissue biopsies were obtained from healthy adult volunteers with no gingival inflammation. The gingival specimens were de-epithelialized with a scalpel for the exclusion of most of the keratinocytes resident in the gingival tissue [33]. In brief, the connective tissue was grinded and then washed several times with PBS (Lonza) and subsequently cultured using TheraPEAK™MSCGM-CD™ BulletKit serum-free, chemically defined (MSCGM-CD) medium for the growth of human MSCs (Lonza) [34]. The medium was changed twice a week, and cells spontaneously migrating from the explant fragments after reaching about 80% of confluence were detached using Triple Select (Lonza).

#### Morphological analysis

hGMSCs at passage 2 were stained with toluidine blue and observed with a Leica DMIL10 (Leica Microsystem, Milan, Italy) inverted light microscope; images were captured using a Leica EC3 digital camera apparatus [35].

#### Scaffold cell performance evaluation

The scaffold morphology, pore size, and cellular attachment were evaluated using scanning electron microscopy (SEM). hGMSCs were seeded onto the 3D PLA scaffold and incubated at 37° for 24 h. They were subsequently fixed for 30 min with 2.5% (v/v) glutaraldehyde in 0.1 M sodium cacodylate and dehydrated in a graded series of ethanol. Afterward, the samples were mounted onto metallic stubs with carbon tape and then sputter-coated with gold using an Emitech K550 sputter coater [36]. The design scaffold “C” (Fig. 1c) had been chosen to be studied.

#### Cytotoxicity of degradation byproducts

The scaffolds were immersed in PBS (0.01 M, pH 7.4), with 0.01 M of ascorbic acid, in a ratio of 6:100 scaffold for in vitro degradation. An aliquot of PBS from degrading PLA scaffolds of the various groups was removed at days 1, 7, 14, 28, 56, and 112 to generate an extract for cytotoxic evaluation of the degradation byproducts. The cellular response to the eluates was evaluated using hGMSCs.

Cells were plated and grown to 80% confluency before initiating the assays. Once at confluence, the eluates were combined with hGMSC cell culture media (in one of three ratios: 1:99, 10:90, and 50:50) and added to hGMSCs that were cultured on tissue culture polystyrene well plates. The eluates and cell culture media solutions were not further adjusted for pH or osmolarity. Cultured cells were exposed to extract and media solution at 37 °C and 5% CO_2_ for 24 h; the cytotoxicity was then quantitatively evaluated with the MTT cell metabolic activity assay. hGMSC culture media without degradation byproducts was used as a control.

The viability of hGMSCs seeded with eluates combined with MSCGM-CD was evaluated in three ratios: 1:99, 10:90, and 50:50. The cellular response was measured by the quantitative colorimetric MTT (3-[4,5-dimethyl-2-thiazolyl]-2,5-diphenyl-2H-tetrazoliumbromide test) (Promega, Milan, Italy) [37]. The cells cultured with MSCGM-CD (Lonza) were used as a control. Cells (2 × 10^3^cells/well) were seeded into a 96-well culture plate with MSCGM-CD medium (Lonza) and, after 24 h of incubation at 37 °C, 15 μl/well MTT was added to the culture medium and the cells were incubated for 3 h at 37 °C [38]. The supernatants were read at a 650-nm wavelength using an ND-1000 NanoDrop Spectrophotometer (NanoDrop Technologies, Rockland, DE, USA). The MTT assay was performed in three independent experiments, with six replicate wells for each experimental time point.

#### hGMSC extracellular vesicles (EVs) isolation

The conditioned medium (CM; 10 mL) after 48 h of incubation were collected from hGMSCs at passage 2. The CM was centrifuged at 3000 × g for 15 min to eliminate suspension cells and debris. For the EVs extraction we used an ExoQuick TC commercial agglutinant (System Biosciences, Euroclone SpA, Milan, Italy). Briefly, 2 mL ExoQuick TC was added to 10 mL of conditioned medium recovered from hGMSCs. The mix was incubated overnight at 4 °C without rotation; one centrifugation step was performed at 1500 × g for 30 min to sediment the EVs and the pellets were resuspended in 200 μL PBS. The detection of EVs whole homogenate protein was used as a confirmation of the presence of release of EVs in hGMSCs.

#### Engineered EVs preparation

The EVs pellet (100 μL) was resuspended with 2 mL PBS, and 2 mL of branched polyethyleneimine solution (PEI; MW 25,000; Sigma-Aldrich, Milan, Italy) (0.05 mg/mL in 0.3 M NaCl) was added to the EVs suspension in PBS and the mixture was incubated for 20 min at room temperature. The suspension was then centrifuged at 4000 rpm for 15 min and the supernatant was removed to get rid of the excess PEI. The precipitate was resuspended in 2 mL PBS. The engineered EVs (PEI-EVs) suspension was characterized using dynamic light scattering (DLS) experiments and ζ-potential measurements.

To evaluate cytotoxicity of the PEI-EVs on hGMSCs, vesicles were incubated with Alexa Fluor 488 Wheat Germ Agglutinin (Life Technologies, Milan, Italy) for 10 min at 37 °C and subsequently analyzed with confocal laser scanning microscopy (CLSM; LSM510 META, Zeiss) after 12 h of incubation.

#### Atomic force microscopy measurements

To evaluate EVs and PEI-EVs surface morphology, atomic force microscopy (AFM) measurements were performed using a Multimode 8 Bruker AFM microscope with Nanoscope V controller (Bruker AXS, Marne La Vallee, France). It is worth highlighting that AFM analyses were performed to visualize the prevailing smallest exosomal objects since it is very difficult to visualize very irregular micrometric surfaces such as those that one could expect for aggregated microvesicles. Nevertheless, several authors have already used this technique to visualize EVs, thus exploiting the mild experimental conditions under which it is possible to visualize them and avoiding the high vacuum of transmission electron microscopy measurements [39]. Silicon cantilever and a RTESPA-300 tip (spring constant = 40 N/m and resonant frequency 300 Hz) were used in a tapping in air mode. The specimen was prepared by dropping a solution of EVs and PEI-EVs on a SiO_2_ wafer followed by air drying at 37 °C for 1 h. The solutions of EVs and PEI-EVs dropcasted onto SiO_2_ water had a different concentration because we wanted to avoid the formation of big aggregates, particularly for the less charged, and consequently more prone to aggregate, PEI-EVs.

#### Dynamic laser light scattering

EVs dispersions were characterized using DLS experiments and ζ-potential measurements using a Brookheaven Zeta Plus.

#### In vitro osteogenesis performance

hGMSCs were seeded at 8 × 10^3^ cells/cm^2^ in MSCGM-CD culture medium (control medium) (Lonza) and in the presence of 3D-PLA, 3D-PLA + EVs, and 3D-PLA + PEI-EVs. Scaffolds were pretreated for 48 h under agitation (MacsMix, Milthenyi, Bologna, Italy) with 5 mL PBS of EVs and PEI-EVs. Evaluation of calcium deposition and extracellular matrix (ECM) mineralization was obtained by Alizarin Red S staining assay performed after 6 weeks. Cells were washed with PBS, fixed in 10% (v/v) formaldehyde (Sigma-Aldrich) for 30 min, and washed twice with abundant dH_2_O prior to the addition of 0.5% Alizarin Red S in H_2_O, pH 4.0, for 1 h at room temperature. After cell incubation under gentle shaking, cells were washed with dH_2_O four times for 5 min. For staining quantification, 800 μL 10% (v/v) acetic acid was added to each well. Cells incubated for 30 min were scraped from the plate, transferred into a 1.5-mL vial, and vortexed for 30 s. The obtained suspension, overlaid with 500 μL mineral oil (Sigma-Aldrich), was heated to 85 °C for 10 min and then transferred to ice for 5 min, carefully avoiding opening of the tubes until fully cooled, and centrifuged at 20,000 × g for 15 min. Then 500 μL of the supernatant was placed into a new 1.5-mL vial and 200 μL of 10% (v/v) ammonium hydroxide was added (pH 4.1–4.5); 150 μL of the supernatant obtained from cultures were read in triplicate at 405 nm by a spectrophotometer (Synergy HT, BioTek, Bad Friedrichshall, Germany) [40].

#### Western blotting

Purified EVs were treated as previously described to protein extraction [41]. Proteins were extracted from EVs and PEI-EVs, from hGMSCs, 3D-PLA+ hGMSCs, 3D-PLA + EVs + hGMSCs, and 3D-PLA + PEI-EVs + hGMSCs after 6 weeks of culture. Proteins were separated on sodium dodecyl sulfate-polyacrylamide minigels and transferred onto PVDF membranes (Immobilon-P Transfer membrane, Millipore, Billerica, MA, USA), blocked with PBS containing 5% nonfat dried milk (PM) for 45 min at room temperature, and subsequently probed at 4 °C overnight with specific CD9 (1:500; Santa Cruz Biotechnology Inc., Santa Cruz, CA, USA), CD63 (1:500; Abcam, Cambridge, UK), CD81 (1:500; Santa Cruz), and TSG101 (1:500; Santa Cruz) for EVs, RUNX2 (1:500; Santa Cruz), BMP2/4 (1:500; Santa Cruz), and β-Actin (1:750; Santa Cruz) for hGMSCs, 3D-PLA + hGMSCs, 3D-PLA + EVs + hGMSCs, and 3D-PLA + PEI-EVs + hGMSCs in 1× PBS, 5% (w/v) nonfat dried milk, 0.1% Tween-20 (PMT). Horseradish peroxidase (HRP)-conjugated goat anti-mouse IgG was incubated as a secondary antibody (1:2000; Santa Cruz) for 1 h at room temperature [42]. The relative expression of protein bands was visualized using an enhanced chemiluminescence system (Luminata Western HRP Substrates, Millipore) and protein bands were acquired and quantified with the ChemiDoc MP System (Bio-Rad, Hercules, CA, USA) and a computer program UVIband-1D gel analysis software (Uvitec, Cambridge, UK), respectively.

#### RNA extraction

Total RNA was isolated from hGMSCs, 3D-PLA + EVs + hGMSCs, and 3D-PLA + PEI-EVs + hGMSCs cultured for 1 week using the Total RNA Purification Kit (Norgen Biotek Corp., Ontario, CA, USA) according to the manufacturer’s protocol. Total RNA was quantified by means of the BioSpectrometer (Eppendorf, Milan, Italy) using μCuvette G1.0 (Eppendorf).

#### Quantitative RT-PCR

To analyze the osteogenic differentiation ability, RUNX2 and BMP2/4 markers were evaluated by means of RT-PCR as previously reported by Diomede et al. [19].

#### RNA sequencing and library preparation

To prepare RNA sequencing libraries the TruSeq RNA Access library kit (Illumina, Inc., San Diego, CA, USA) was used according to the manufacturer’s instructions. Briefly, 50 ng of RNA from each sample was fragmented at 94 °C for 8 min. In a first strand phase, the cDNA was synthesized by using random hexamers and the SuperScript II Reverse Transcriptase (Invitrogen, Milan, Italy) at 25 °C for 10 min, 42 °C for 15 min, and 70 °C for 15 min.

In a second strand of cDNA synthesis, the RNA templates were removed and a second replacement strand was generated by dUTP internalization to produce double-strand cDNA. Afterwards, to purify the blunt-ended cDNA, AMPure XP beads (Beckman Coulter, Brea, CA, USA) were used. The 3′ ends of the cDNA were adenylated to permit the adaptor ligation in the subsequent step. After that, the libraries were purified with AMPure XP beads. A first PCR amplification step was performed to enrich those fragments of DNA that have adaptors on both ends, and also to enhance the quantity of DNA in the library (15 cycles of 98 °C for 10 s, 60 °C for 30 s, and 72 °C for 30 s). The library has been validated using the Agilent Technologies 2100 Bioanalyzer. After that, 200 ng of each DNA library was combined and the first hybridization step was performed using exome capture probes according to a standardized protocol (18 cycles, starting at 94 °C, and then decreasing by 2 °C for every cycle). To eliminate nonspecific binding, magnetic beads coated with streptavidin were used to capture probes hybridized to the target regions. Another capture round with streptavidin-coated beads was performed, followed by two heated washes to discharge the nonspecific binding from the beads. Then, the enriched libraries were eluted from the beads and were ready for a second cycle of hybridization. This hybridization step was necessary to obtain a wide specificity of regions of capture. After that, the libraries were purified through the AMPure XP bead, and amplified according to the protocol (10 cycles; incubation at 98 °C for 10 s, incubation at 60 °C for 30 s, and incubation at 72 °C for 30 s), followed by a purification step. Libraries were quantified by the qPCR using KAPA Library Quantification Kit—Illumina/ABI Prism_ (Kapa Biosystems, Inc., Wilmington, MA, USA) and certified with the Agilent High Sensitivity Kit on a bioanalyzer. The size of the DNA fragments has been set in a range 200–650 bp and peaked around 250 bp. Libraries were normalized to 12pM and subjected to cluster, and single read sequencing was executed for 150 cycles on a MiSeq instrument (Illumina) following the protocol guidelines. The produced libraries were loaded for clustering on a MiSeq Flow Cell v3 and then sequenced with a MiSeq Instrument (Illumina) [43]. The cluster density validation had been executed by the software of the instrument throughout the run.

#### Next-generation sequence data processing

Data obtained by next-generation sequencing (NGS) analysis were processed. Specifically, the sequence reads were subjected to the demultiplexing process to have a separation of the sequence reads in different files for each index tag/sample by using the CASAVA algorithm (CASAVA, version 1.8.2; Illumina, Inc., San Diego, CA, USA). Then, for the alignment of sequences, the RNA-Seq Alignment version 1.0.0 (Illumina) and the reference sequence “*Homo sapiens* UCSC hg19” for the read mapping the TopHat 2 (Bowtie 1) were used. The fragments per kilobase of exon per million fragments mapped (FPKM) values were calculated for each sample using the normalized read counts for each annotated gene: ([1000 × read count] / [number of gene covered bases × number of mapped fragments in million]). Unmapped reads were deleted, preserving only read pairs with both reads aligned to the reference sequence “*Homo sapiens* UCSC hg19.” The comparison between two different specimens was performed by a scatter plot of the log_2_ of the FPKM.

#### Statistical analysis

Statistical analysis was accomplished using analysis of variance (ANOVA) and Tukey’s post-hoc analysis (*p* < 0.05). The statistical data on the read counts were carried out with the Cufflinks Assembly&DE package version 2.0.0 to establish the proportion of differentially expressed genes for a *q* value < 0.05. The gene ontology (GO) analysis of the genes differentially expressed between experimental groups were performed by the free tools “Gene Ontology Consortium” (available online at http://www.geneontology.org/).

#### Animals

Male Wistar rats weighing 300–350 g were used for this experiment. Animals were acquired from Harlan, Milan, Italy, and housed in individually ventilated cages and maintained under 12-h light/dark cycles at 21 ± 1 °C and 50–55% humidity with food and water ad libitum.

#### Scaffold grafting

To implant the scaffold, rats were first anesthetized with a combination of tiletamine and xylazine (10 mL/kg, intraperitoneally). Afterwards, the implant site was prepared with iodopovinone (Betadine) after trichotomy. Following a median sagittal incision of about 2.5 cm from the occipital region, a total thickness cut was applied; the calvaria was then exposed in the frontal area and in the parietal areas. The circular section bone receiving site, with a diameter of 5 mm and a height of 0.25 mm, was injured by means of a rotary instrument at a controlled speed (trephine milling machine, Alpha Bio-Tec, HTD Consulting S.r.l., Siena, Italy) under constant irrigation of a physiological solution.

For their texture and flexibility, 3D-PLA, 3D-PLA + hGMSCs, 3D-PLA + EVs, 3D-PLA + PEI-EVs, 3D-PLA + EVs + hGMSCs, and 3D-PLA + PEI-EVs + hGMSCs were easily inserted in contact with bone tissue to cover the damaged area. The skin flap was then sutured with Caprosyn 6-0 synthetic monofilament adsorbable sutures (Covidien AG, Neuhausen am Rheinfall, Switzerland) using interrupted points. Standard feeding and hydration were maintained as a constant throughout the postoperative phase.

The design scaffold “C” was chosen to be implanted in the host tissue.

#### Experimental design

Rats were randomly distributed into the following groups (*n* = 24 total animals):

1. 3D-PLA (*n* = 4): rats subjected to scraping of the cortical calvarial bone tissue and implant of 3D-PLA;
2. 3D-PLA + hGMSCs (*n* = 4): rats subjected to scraping of the cortical calvarial bone tissue and implant of 3D-PLA + hGMSCs;
3. 3D-PLA + EVs (*n* = 4): rats subjected to scraping of the cortical calvarial bone tissue and implant of 3D-PLA + EVs;
4. 3D-PLA + EVs + hGMSCs (*n* = 4): rats subjected to scraping of the cortical calvarial bone tissue and implant of 3D-PLA + EVs + hGMSCs;
5. 3D-PLA + PEI-EVs (*n* = 4): rats subjected to scraping of the cortical calvarial bone tissue and implant of 3D-PLA + PEI-EVs.
6. 3D-PLA + PEI-EVs + hGMSCs (*n* = 4): rats subjected to scraping of the cortical calvarial bone tissue and implant of 3D-PLA + PEI-EVs + hGMSCs.

After 6 weeks the animals were euthanized by intravenous administration of Tanax (5 mL/kg body weight) and their calvariae were processed for morphological analysis.

For each experiment we used 2 × 10^6^ hGMSCs stained with PKH-26 and EVs and PEI-EVs stained with PKH-67, according to the Sigma procedures.

The specimens were fixed for 72 h in 10% formalin solution, dehydrated in ascending graded alcohols, and embedded in LR White resin (Sigma-Aldrich). After polymerization, undecalcified oriented cut sections of 50 μm were prepared and ground down to about 30 μm using the TT System (TMA2, Grottammare, Italy).

The sections were analyzed before staining with CLSM (LSM510 META, Zeiss) and, after a double-staining procedure with methylene blue and fuchsin acid solutions, they were observed under a light microscope [44].

The investigation was carried out by means of a bright-field light microscope (Leica Microsystem) connected to a high-resolution digital camera DFC425B Leica (Leica Microsystem). Histomorphometry, shown as the percentage of the newly formed bone, was carried out using a digitizing pad (Matrix Vision GmbH, Oppenweiler, Germany) and a histometry software package with image capturing capabilities (Image-Pro Plus 4.5, Media Cybernetics Inc., Immagini & Computer Snc, Milano, Italy).

Three-dimensional reconstruction was obtained by means of ZEN2 software (Zeiss). Data and statistical analysis were performed with the Statistical Package for Social Science (SPSS, v.21.0, IBM Analytics, Armonk, NY, USA).

To confirm the presence of hGMSCs in the sample groups 3D-PLA + hGMSCs, 3D-PLA + EVs + hGMSCs, and 3D-PLA + PEI-EVs + hGMSCs, the sections were rehydrated for 30 min and subsequently stained with human anti-ANA (1:200; Merck Millipore, Milan, Italy) and observed with CLSM (LSM800, Zeiss).

#### Computed tomography (CT)

CT was used to evaluate the bone repair. CT analysis of calvariae was performed on a Siemens Somatom Definition AS operated at 70 kV/350 mA. The thickness of acquisition was 0.6 mm with a range of reconstruction of 0.1 mm.

## Results

### 3D PLA scaffold characterization

**Fig. 2:**
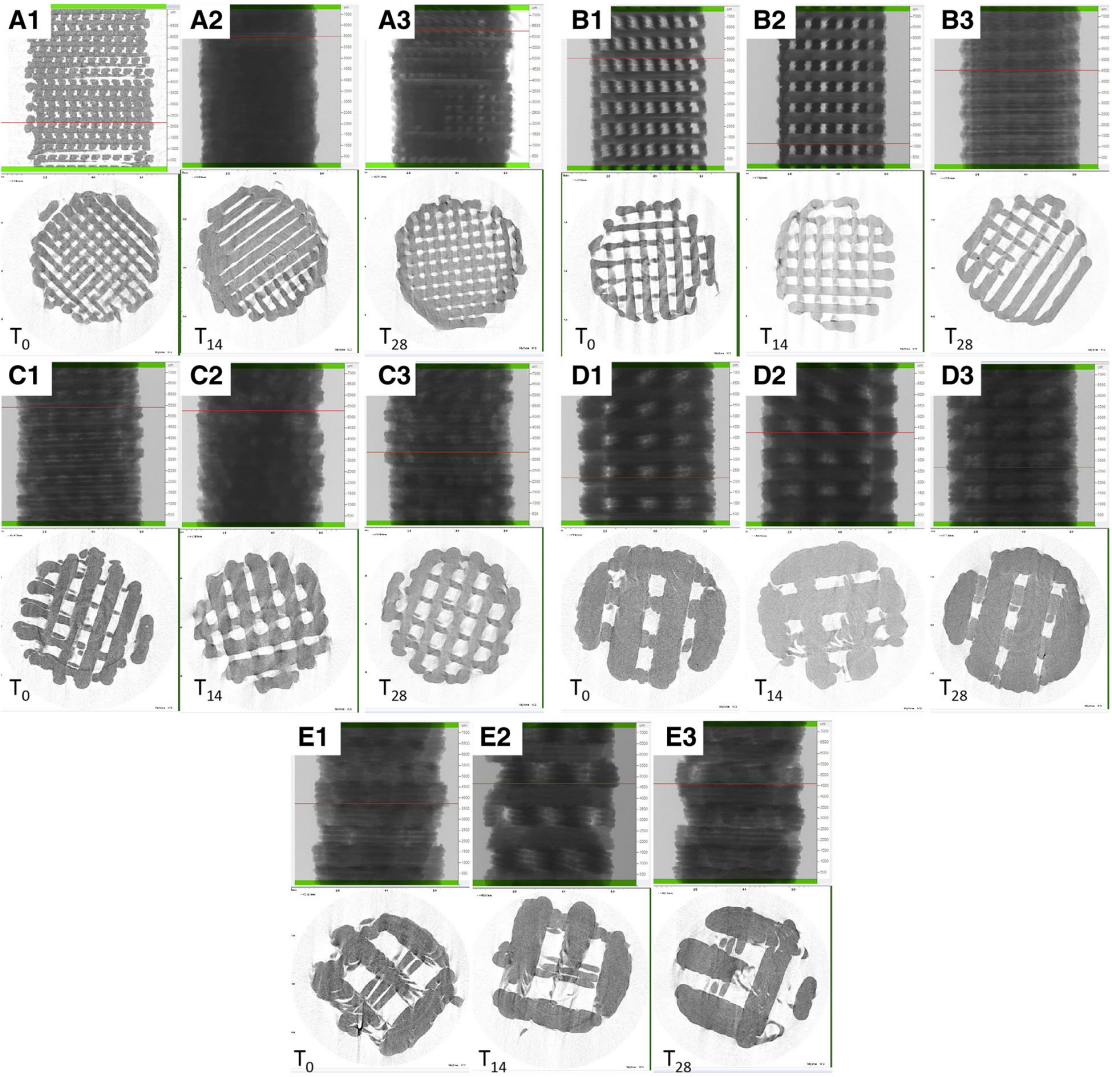
Microcomputed tomography three-dimensional rendering and evaluation of porosity, pore size, and wall thickness at different degradation time point. Scaffolds with **a** design A, **b** design B, **c** design C, **d** design D, and **e** design E at T_0_, T_14_, and T_28_. mCT values at T_0_ for porosity, trabecular thickness (TT), and trabecular separation (TS)

The 3D design of the five different generated scaffolds are shown in Fig. 1a–e. Surface morphology, pore and trabecular dimensions, and total porosity of the 3D printed scaffolds were characterized using SEM (Fig. 1a1–e1) and mCT (Fig. 2). During printing, the scaffolds exhibited a reduction in real pore size and trabecular separation compared to their design, resulting in a concurrent reduction in total porosity. There was also a reduction compared to planned values for design D and E (Fig. 2). Furthermore, scaffolds with larger filament/pore dimensions showed a mild layering within each trabecular area (Fig. 1f). Specific bands of RUNX2 were present in all samples of different design, while a higher expression of osteogenic-related marker is clearly visible in hGMSCs cultured with design scaffold C (Fig. 1g).

### 3D PLA in vitro degradation

**Fig. 3:**
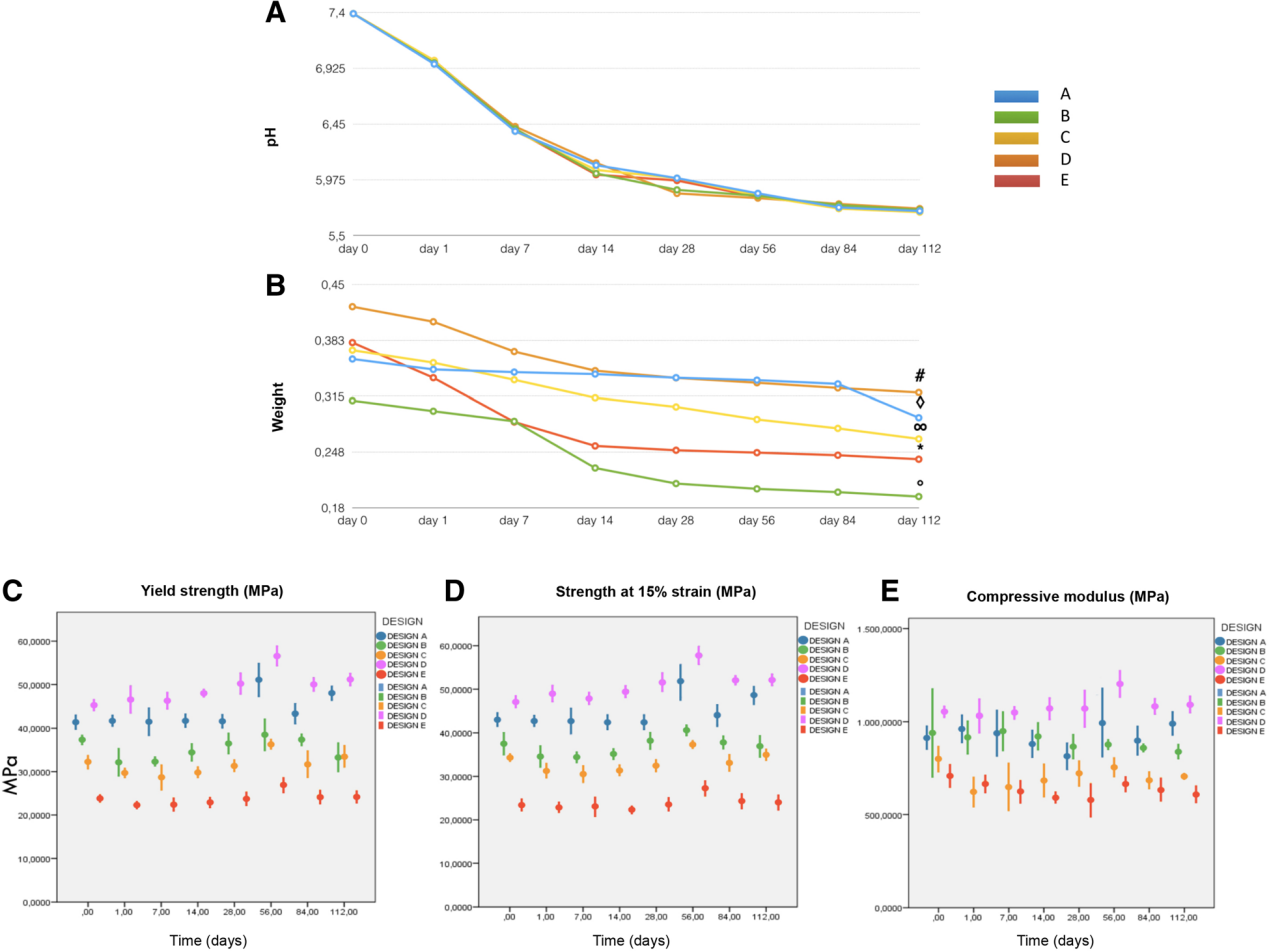
**a** Graph showing pH decrease during 3D-PLA degradation at different time points. **b** Graph showing weight loss during 3D PLA degradation at different time points. **c** Yield strength with significantly higher values for designs D over B, C, and E at day 0 and after 112 days. Design B showed a significant decrease over time. **d** Mean strength at 15% strain (±SD) for the tested groups for each time point. 3D PLA design D values were statistically higher than B, C, and E; differences between groups were confirmed after degradation except for design A which showed higher stability over time. **e** Compressive modulus with significantly higher values for design D over designs C and E at day 0 and after 112 days. Design B showed a significant decrease over time

Degradation in ascorbic acid solution was measured at day 112 by the following changes in mass and pH. The lowest values in pH were recorded at day 112 for all groups (Fig. 3a). Mass loss increased throughout the study, with the biggest increase after 28 days (Fig. 3b). The various scaffold designs generally maintained their compressive mechanical properties during degradation in vitro. For strength at 15% of strain at T_0_, design E had significantly lower values compared with the other four groups, while design D values were statistically higher than those obtained for designs B, C, and E. On the other hand, design A preserved a significantly higher strength at the end of degradation compared with designs B and C at the same time point. Designs A and C showed the best performances in terms of strength after degradation (Fig. 3c). In terms of yield strength at T_0_, design D showed significantly higher values compared with designs B, C, and E; specimens from design E had significantly lower values compared with the other four scaffolds. On the other hand, design B showed a decrease in terms of yield strength (Fig. 3d). As far as the compressive modulus at T_0_ is concerned, design D showed the highest values, with a statistically significant difference compared with design C. After 112 days, this group also showed a significant difference compared with design B that showed a decrease in compressive modulus (Fig. 3e).

### MTT evaluation of cytotoxicity of degradation byproducts

The metabolic activity of cells exposed to the extract of degrading PLA scaffolds was statistically different from the control group for all PBS extract concentrations, scaffold design, and degradation times. However, designs A and C showed no difference in terms of cell metabolic activity between T_1_ and T_112_, irrespective of extract/cell ratio. Designs B, D, and E showed a difference between T_1_ and T_112_ for all the ratios. Positive peaks in cell activity within a degradation time point could not be confirmed for the three extract/cell ratios used, as reported in more detail by MTT graphs (see Additional file 1).

### 3D PLA and hGMSC interaction

Since synthesis design C demonstrated the best features in terms of mechanical and chemical properties, this was used to analyze the interaction of hGMSCs with 3D PLA. Morphological analysis by SEM demonstrated numerous extensions of cytoplasmic processes which enabled cellular anchorage (see Additional file 2, section A). Cells spread and extended on the uneven surface and across the filaments to create extended contact areas between them, organizing a multilayer covering the 3D substrate. The biomaterial surface covered with hGMSCs is evident when compared with 3D PLA without cells (see Additional file 2, inset in section A). At high magnification, an increased number of cellular bridges were demonstrated (see Additional file 2, section B). The scaffold material did not induce visible changes in cellular morphology.

### EV and PEI-EV characterization

**Fig. 4:**
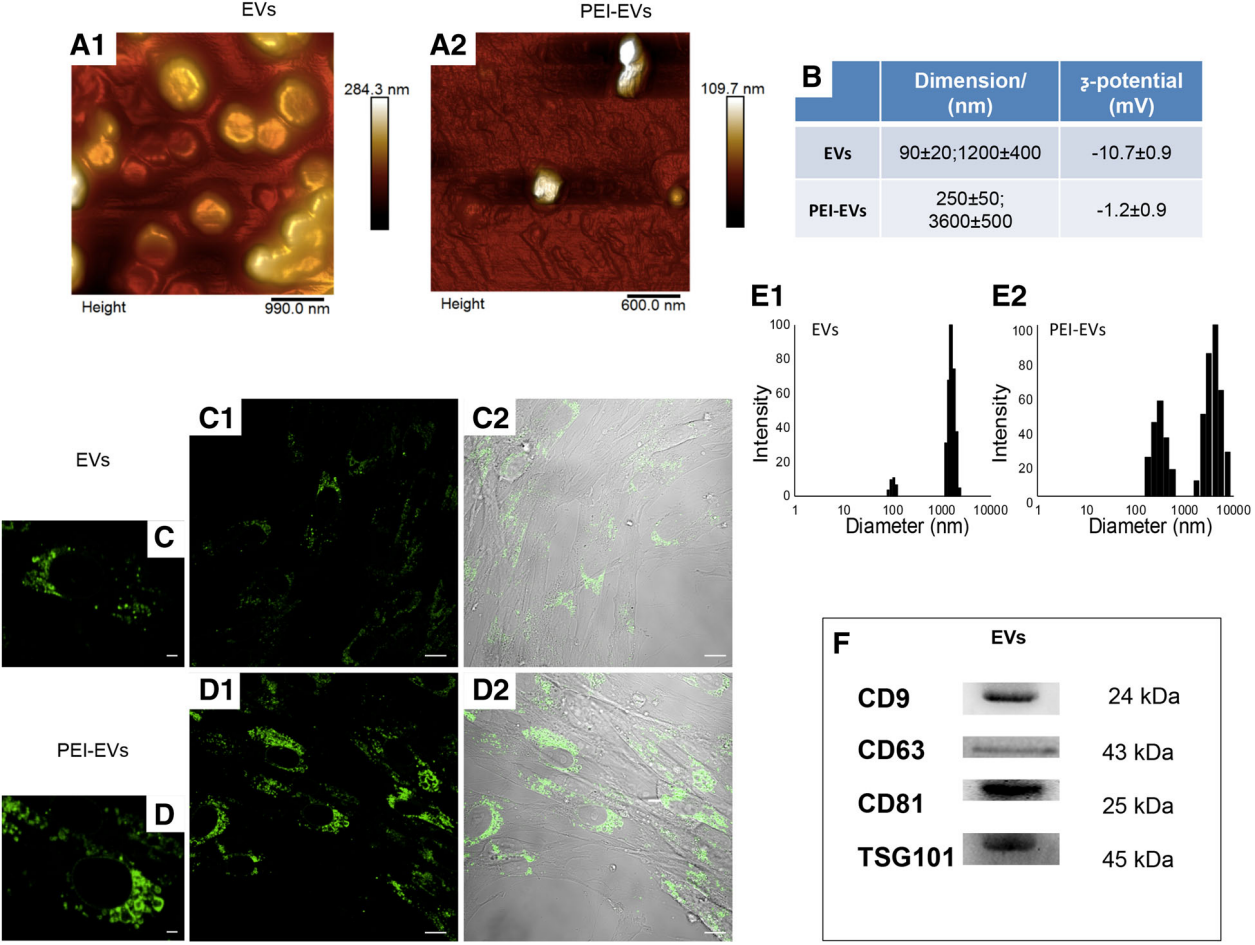
Extracellular vesicles (EVs) and polyethyleneimine (PEI)-engineered EVs (PEI-EVs) characterization. **a** Tapping mode topographic AFM image showing round morphology of EVs and PEI-EVs. **b** Average size and ζ-potential of EVs and PEI-EVs. High-magnification images (40×) of hGMSCs cured with **c** EVs and **d** PEI-EVs panel 1: hGMSCs cultured in the presence of WGA-stained EVs and PEI-EVs observed with confocal laser scanning microscopy after 24 h; panel 2: bright field of hGMSCs/WGA-stained EVs and PEI-EVs. **e** DLS size distribution histograms of EVs and PEI-EVs. **f** Western blot showed the positivity for CD9, CD63, CD81, and TSG101. Scale bars in **c,d** = 10 μm

The DLS analysis shows the presence of a heterogeneous population of EVs, spanning from 100 to 1200 nm. In particular, two main dimensional populations could be identified, the average diameter of the first population being 93 ± 24 nm and that of the second population being 1200 ± 400 nm (Fig. 4b, e1). Both populations increased in dimension after the addition of PEI, with the final size being 250 ± 50 nm and 3600 ± 500 nm, respectively (Fig. 4b, e2). The EV suspension had a ζ-potential of −10.7 ± 0.9 mV, whereas after the addition of the cationic polyelectrolyte PEI, there was an increase to −1.2 ± 0.9 mV coating (Fig. 4b). Although these size increases are particularly high for a simple coating of PEI, they also show the occurrence of a PEI coating. Indeed, the decrease in ζ-potential, consequent to the PEI adsorption (see below), may favor the formation of small aggregates.

EVs and PEI-EVs were also analyzed by AFM. Figure 4a1 highlights the presence of a large number of globular EVs of different dimensions with a central depression, thus confirming previous reports on the shape of EVs [39] as well as DLS data. Some debris or aggregated vesicles were also observed. Very interestingly, the surface of EVs appeared relatively smooth. On the other hand, the specimen of PEI-EVs (Fig. 4a2) shows objects of homogeneous dimensions, with no central depression, and characterized by a less smooth surface with respect to pure EVs; likely these latter findings confirm the adsorption of PEI onto the EVs surface.

hGMSCs were incubated with EVs and PEI-EVs stained with WGA Alexa Fluor 488. After 2 days, CLSM images were captured and the viability was estimated to be in the range 80–90%. Very interestingly, the number of vesicles in the cells is higher when the PEI-EVs (Fig. 4d) rather than nonengineered EVs are used (Fig. 4c), indicating a higher capacity of EVs to enter the cells when coated. At high magnification, the vesicles in the cytoplasmic compartment are clearly visible (Fig. 4c, d). Western blot analysis performed on EVs showed a positivity for CD9, CD63, CD81, and TSG101 molecules (Fig. 4f).

### Osteogenic differentiation

**Fig. 5:**
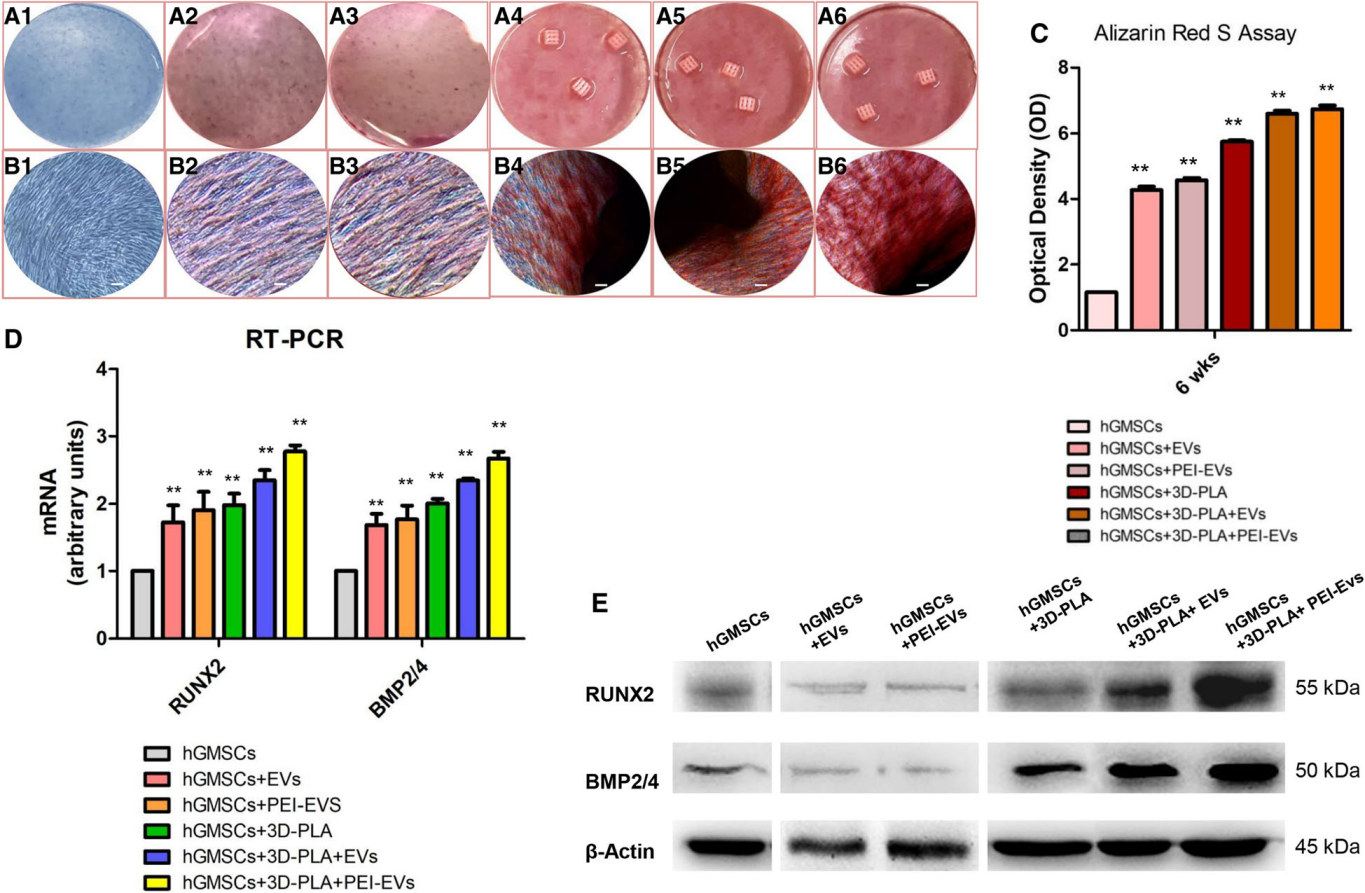
In vitro osteogenic induction. Human gingival mesenchymal stem cells (hGMSCs), maintained under osteogenic conditions for 6 weeks, were stained using Alizarin Red S solution. **a** Macro photographs. **b** Photographs obtained at light microscopy. Scale bars = 5 μm. **c** Graph optical density. **d** RT-PCR of osteogenic related markers. **e** Western blots of RUNX2 and BMP2/4; β-Actin was used as the housekeeping protein. **p < 0.01, versus hGMSCs. EVs, extracellular vesicles; PEI, polyethyleneimine

Low-magnification photographs were used to verify the Alizarin Red S staining (Fig. 5a); meanwhile, light microscopy imaging was used to highlight cell osteogenic differentiation at high magnification under four different culture conditions (Fig. 5b). The best results in terms of production of calcium deposits were shown by hGMSCs cultured in the presence of 3D-PLA + EVs and 3D-PLA + PEI-EVs.

Data were quantified using spectrometric analysis after 6 weeks of osteogenic induction (Fig. 5c). RT-PCR was performed to analyze changes in RUNX2 and BMP2/4 gene expression in all groups after 6 weeks of culture. We observed increases in RUNX2 and BMP2/4 mRNA expression in the 3D-PLA + EVs and 3D-PLA + PEI-EVs group, respectively, which were significantly higher than the control group (Fig. 5d). Western blotting indicated consistent findings for RUNX2 and BMP2/4 protein expression (Fig. 5e).

### Global transcriptome

The transcriptome of hGMSCs, 3D-PLA + EVs + hGMSCs, and 3D-PLA + PEI-EVs + hGMSCs was investigated using high-throughput sequencing with an Illumina MiseqDx. Statistical analysis revealed that 31 genes were differentially expressed between the examined groups. Specifically, the analysis of these genes expressed among hGMSCs, 3D-PLA + EVs + hGMSCs, and 3D-PLA + PEI-EVs + hGMSCs was performed by inserting the selected genes on the online database “Gene Ontology Consortium” identify a putative GO class. According to GO analysis, 31 genes involved in “regulation of ossification” and “ossification” were upregulated in the 3D-PLA + PEI-EVs + hGMSCs group compared to the hGMSCs group (false discovery rate (FDR) = 8.14 × 10^−35^). Moreover, among these genes, 16 genes were upregulated in the 3D-PLA + EVs + hGMSCs group compared with the hGMSCs group (FDR = 3.03 × 10^−15^) (see Additional file 3A and Additional file 4: Table S1).

GO analysis also showed that 19 genes belonging to the family GO “regulation of osteoblast differentiation” and “osteoblast differentiation” were upregulated in the 3D-PLA + PEI-EVs + hGMSCs group compared with the hGMSCs group (FDR = 2.56 × 10^−15^). Among these genes, 10 genes were upregulated in the 3D-PLA+ EVs + hGMSCs group compared with the hGMSCs group (FDR = 4.42 × 10^−14^) (see Additional file 3B and Additional file 4: Table S2). Finally, the comparison between the two big GO families (ossification and osteoblast) showed 19 genes that were common to the both families.

Moreover, 9 genes reported to be upregulated during osteogenesis were more expressed in 3D-PLA + PEI-EVs + hGMSCs compared with hGMSCs [45] (see Additional file 4: Table S3 and Additional file 5). Furthermore, some genes reported to be involved in osteoblast differentiation through transforming growth factor (TGF)-β signaling were upregulated in 3D-PLA + PEI-EVs + hGMSCs (TGFBR1, SMAD1, BMP2, MAPK1, MAPK14, and RUNX2) [46] (see Additional file 4: Table S3 and Additional file 5).

In addition, gene expression profiles of adhesion molecules and ECM were evaluated in the 3D-PLA + PEI-EVs + hGMSCs group compared with control cells by NGS analysis. There were a total of 20 differentially expressed genes, including 9 upregulated genes and 11 downregulated genes (see Additional file 4: Table S4 and Additional file 6). As shown in Additional file 6, genes that encode integrin, basement membrane laminins, membrane proteins that mediate cell-to-cell and cell-to-matrix interactions, and inhibitor of the matrix metalloproteinases were upregulated in 3D-PLA + PEI-EVs + hGMSCs. In parallel, genes that encode for cell-cell adhesion and for the ECM constituents of basement membranes were downregulated in 3D-PLA + PEI-EVs + hGMSCs.

### 3D PLA in vivo evaluation

**Fig. 6:**
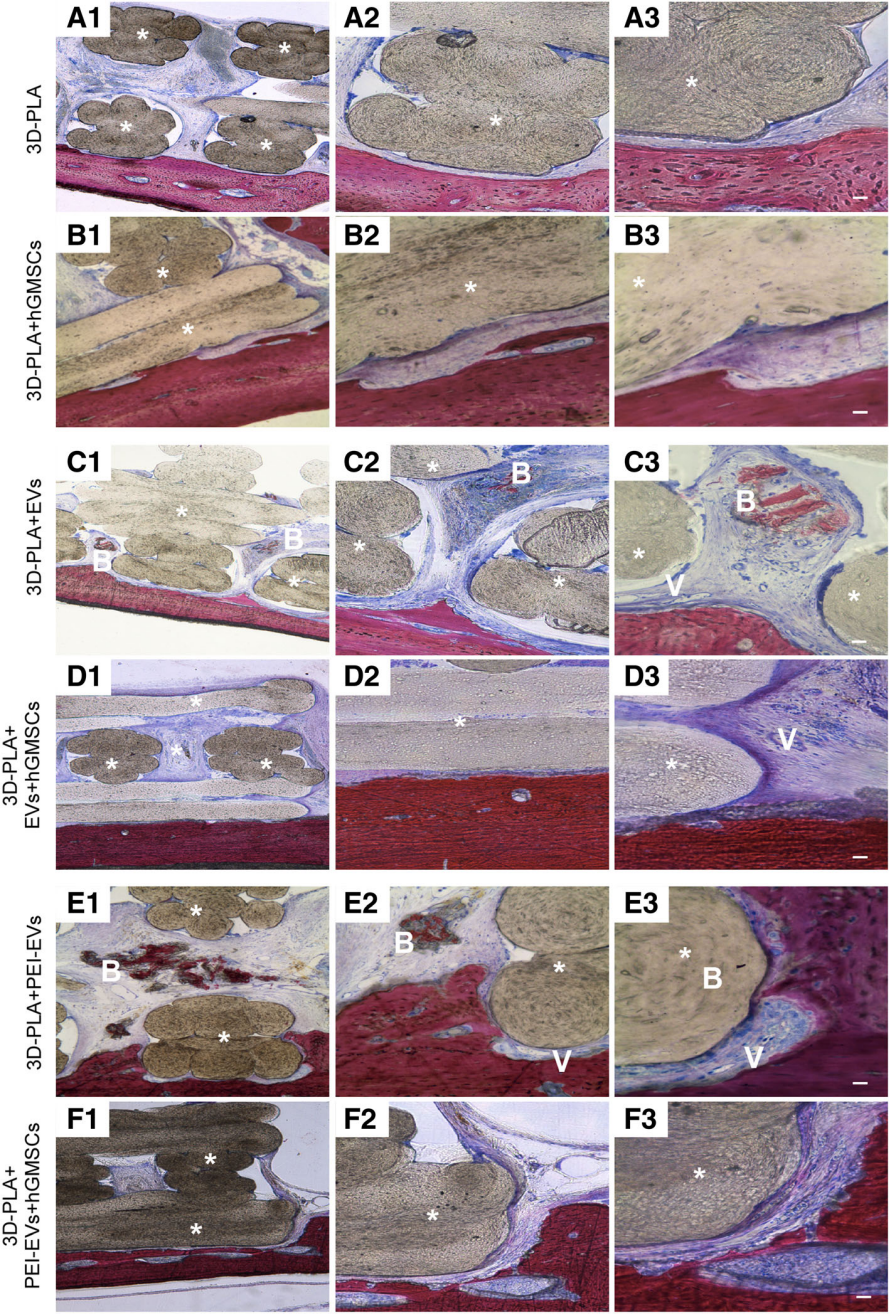
Histological evaluation. Samples harvested at 6 weeks after the calvarial defects were transplanted with **a** 3D-PLA scaffold or **b** 3D-PLA + human gingival mesenchymal stem cells (hGMSCs). Left panels (1): The images at low magnification (4×) showed 3D-PLA and 3D-PLA + hGMSCs integrated smoothly with the host tissue. Middle panels (2): High-magnification images (10×) showing the contact area between 3D-PLA and 3D-PLA + hGMSCs with bone calvaria grafted at 6 weeks postsurgery. Right panels (3): Images obtained at 40× objective showing the connective tissue between 3D-PLA and 3D-PLA + hGMSCs and bone host tissue. Samples harvested at 6 weeks after the calvarial defects was transplanted with **c** 3D-PLA + extracellular vesicles (EVs) scaffold or **d** 3D-PLA + EVs + hGMSCs. Left panels (1): The images at low magnification (4×) showed 3D-PLA + EVs and 3D-PLA + EVs + hGMSCs integrated smoothly with the host. Middle panels (2): High-magnification images (10×) showing the new bone formation stained with acid fuchsin in both samples grafted at 6 weeks postsurgery. Right panels (3): Images obtained at 40× objective showed a zone with new mineralized matrix inside 3D PLA scaffold for both samples. In particular, in the 3D-PLA + EVs **(c3)** sample in the contact zone some blood vessels are valuable. Samples harvested at 6 weeks after the calvarial defects was transplanted with **e** 3D-PLA + polyethyleneimine (PEI)-EVs scaffold or **f** 3D-PLA + PEI-EVs + hGMSCs. Left panels (1): The images at low magnification (4×) showed 3D-PLA + PEI-EVs and 3D-PLA + PEI-EVs + hGMSCs integrated smoothly with the host. Middle panels (2): High-magnification images (10×) showing the new bone formation stained with acid fuchsin in both samples grafted at 6 weeks postsurgery. Right panels (3): Images obtained at 40× objective showed new bone formation inside the scaffold structure. In particular, in 3D-PLA + PEI-EVs there were numerous blood vessels present around the new bone deposition area. Scale bars = 10 μm. *, scaffold; V, vessels; B, new bone. Acid fuchsin-toluidine blue staining

Overall, the scaffolds harvested after 6 weeks of implantation in calvaria of rats contained 3D PLA scaffold infiltrated or not with hGMSCs. Histological samples after staining with acid fuchsin and methylene blue solution showed a different response to various substrates. In 3D-PLA samples, ECM without signs of mineralization was observed (Fig. 6a), while in 3D-PLA + hGMSCs samples the integration process had started, with deposition of new ECM (Fig. 6b).

More host tissue ingrowth in the implant site was observed in 3D-PLA + EVs and 3D-PLA + EVs + hGMSCs when compared with the 3D-PLA and 3D-PLA + hGMSCs (Fig. 6).

Abundant ECM and nodules of new bone formation stained with acid fuchsin were present in both samples, while blood vessels formation was visible in 3D-PLA + EVs samples (Fig. 6d) when compared with 3D-PLA + EVs (Fig. 6c).

In samples grafted with 3D-PLA + PEI-EVs and 3D-PLA + PEI-EVs + hGMSCs there was a significant difference in host tissue response. New bone deposition and ECM areas, blood vessels formation, and osteoblast-like cells are valuable in bone defects grafted with 3D-PLA + PEI-EVs + hGMSCs (Fig. 6f). In the 3D-PLA + PEI-EVs samples, numerous bone nodules were visible as well as various blood vessels of different dimensions indicating a new vascular network formation (Fig. 6e).

**Fig. 7:**
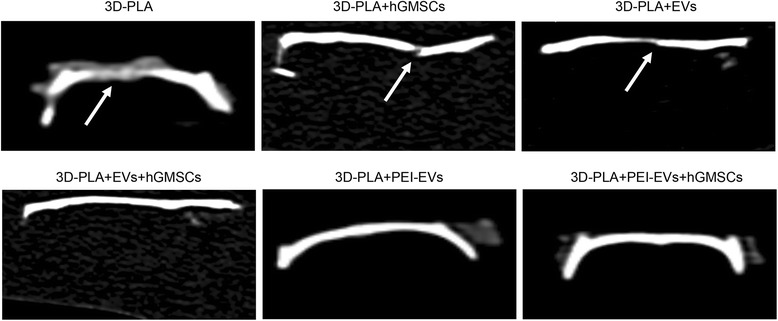
CT analysis showing bone damage was still present in the 3D-PLA, 3D-PLA + human gingival mesenchymal stem cells (hGMSCs), and 3D-PLA + extracellular vesicles (EVs) groups. On the contrary, in the 3D-PLA + EVs + hGMSCs, 3D-PLA + polyethyleneimine (PEI)-EVs, and 3D-PLA + PEI-EVs + hGMSCs groups the complete repair of the calvarial defect was shown. Arrows indicated bone defects

CT evaluation showed that bone damage was still present in the 3D-PLA, 3D-PLA + hGMSCs, and 3D-PLA + EVs samples. However, in the 3D-PLA + EVs + hGMSCs, 3D-PLA + PEI-EVs, and 3D-PLA + PEI-EVs + hGMSCs samples, the complete repair of the calvarial defect was visible (Fig. 7).

Histomorphometry analysis showed that newly formed bone represented 12.27%, and the total surface area constituted by ECM and biomaterial residual graft material 87.72%, as reported in Table 2.

**Table 2:** Histomorphometric analysis

Sample	% new bone	% 3D-PLA + ECM
3D-PLA	0	100
3D-PLA/hGMSCs	3.07519116	96.9,248,088
3D-PLA/Evs	0.81936967	99.1806303
3D-PLA/EVs/hGMSCs	1.73274672	98.2672533
3D-PLA/PEI-EVs	12.2733915	87.7266085
3D-PLA/PEI-EVs/hGMSCs	9.71949016	90.2805098

ECM, extracellular matrix; EVs, extracellular vesicles; hGMSCs, human gingival mesenchymal stem cells; PEI, polyethyleneimine

**Fig. 8:**
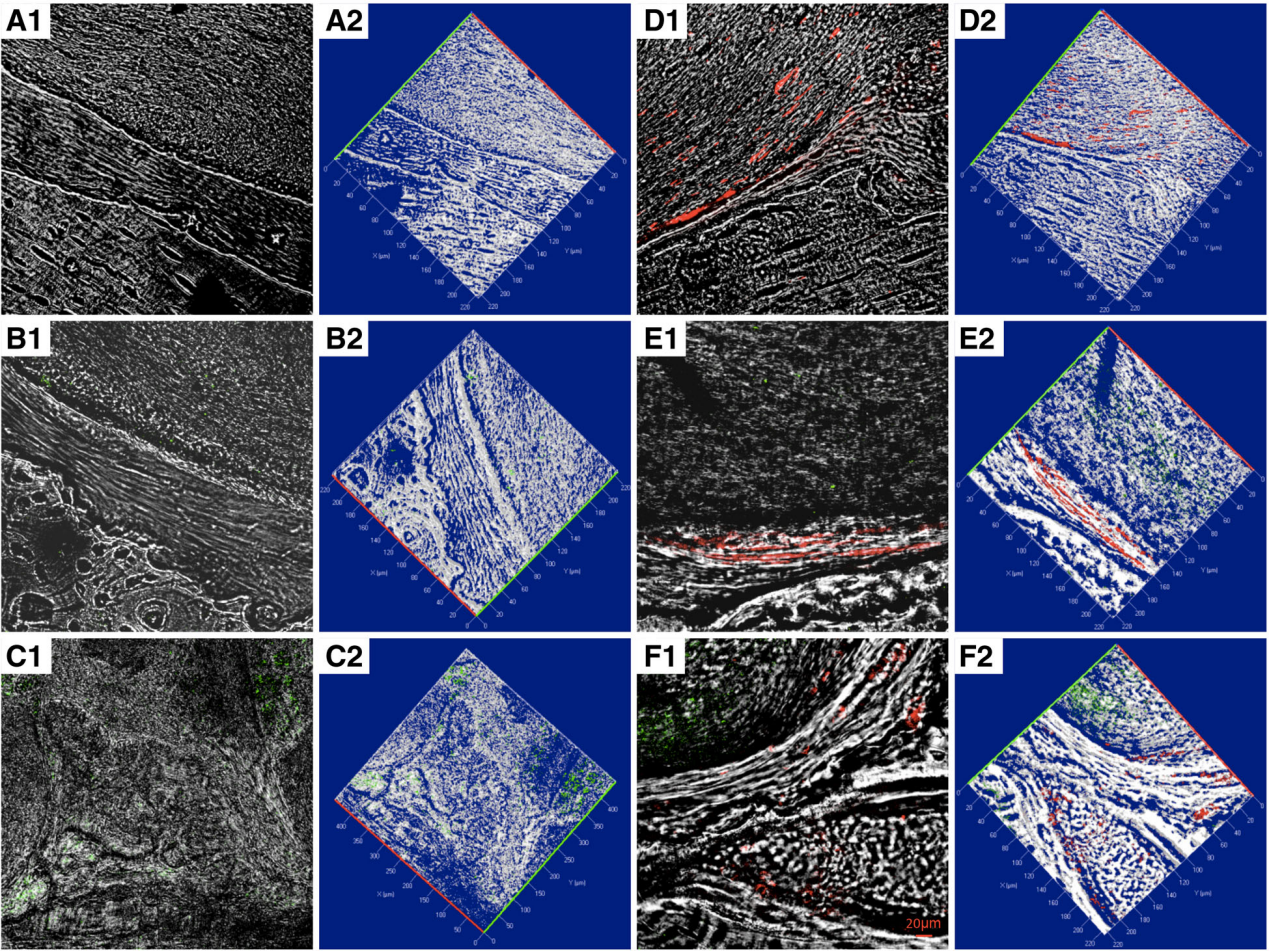
Confocal laser scanning microscopy analysis. **a** Confocal contrast image (left panel 1) of 3D-PLA (gray) grafted in mice calvaria after 6 weeks of implantation, with the corresponding 3D reconstruction image (right panel 2). **b** 3D-PLA (gray) and PKH-67-stained EVs (green) after 6 weeks of in vivo implantation, with the corresponding 3D reconstruction image. **c** 3D-PLA (gray) and PKH-67-stained PEI-EVs (green), with the corresponding 3D reconstruction image. **d** 3D-PLA (gray) and PKH-26-stained hGMSCs (red), with the corresponding 3D reconstruction image. **e** 3D-PLA (gray), PKH-26-stained hGMSCs (red), and PKH-67-stained EVs, with the corresponding 3D reconstruction image. **f** 3D-PLA (gray), PKH-26-stained hGMSCs (red), and PKH-67-stained PEI-EVs, with the corresponding 3D reconstruction image. Scale bars = 20 μm

To demonstrate the presence and the ability for bone regeneration of hGMSCs and EVs in the host tissue, cells were stained with PKH-26 and EVs or PEI-EVs were stained with PKH-67. Confocal analysis further demonstrated the presence of hGMSCs grafted with the 3D-PLA (Fig. 8d1, e1, f1), of EVs (Fig. 8b1, e1) and of PEI-EVs (Fig. 8c1, f1).

3D reconstruction of each microscopy image showed the spatial organization between the scaffold, cells, and EVs or PEI-EVs in the grafted site (Fig. 8a2–f2).

**Fig. 9:**
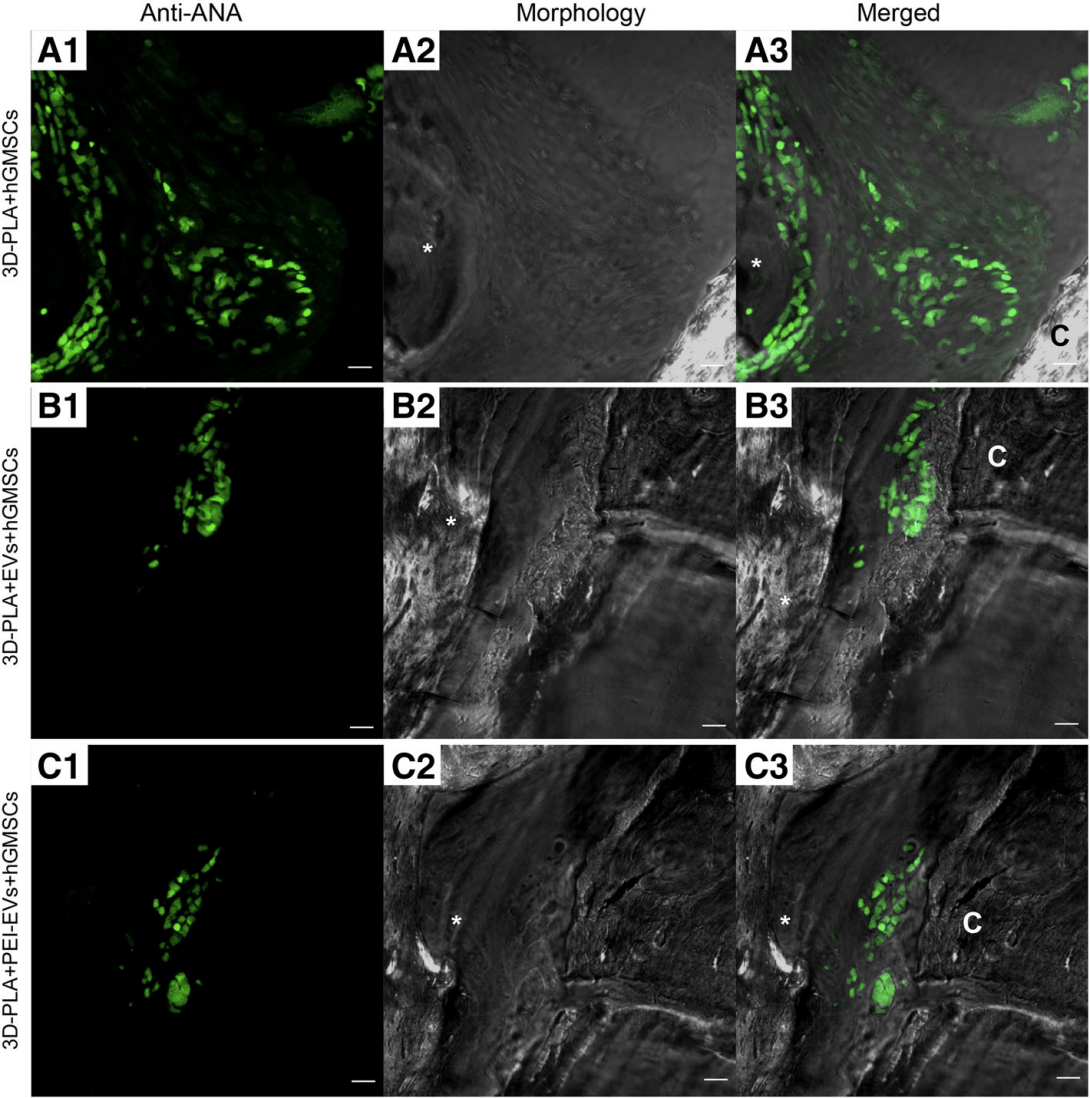
Confocal laser scanning microscopy analysis. Left panels: Human gingival mesenchymal stem cells (hGMSCs) anti-ANA positive nuclei in **a** 3D-PLA + hGMSCs, **b** 3D-PLA + extracellular vesicles (EVs) + hGMSCs, and **c** 3D-PLA + polyethyleneimine (PEI)-EVs + hGMSCs (green fluorescent). Middle panels: Confocal contrast image of 3D-PLA (gray) grafted in mice calvaria after 6 weeks of implantation. Right panels: Merged images of the two abovementioned channels. Scale bars = 10 μm. *, scaffold; C, rat calvaria

Human GMSCs nuclei labeled with human anti-ANA green fluorescent conjugate are clearly visible in the rat calvaria, confirming the presence of living transplanted cells (Fig. 9).

## Discussion

Three-dimensional printing now allows fabrication of complex scaffold designs with different interconnections, porosity, and pore shape that were previously difficult to build [47, 48]. In this study, five innovative original designs of 3D-PLA scaffolds have been printed and their filament/pore sizes have been characterized. The broad range of tested porosities represents the novelty of this work. These scaffolds were then doped with hGMSCs-derived extracellular vesicles and tested for their ability to regenerate bone defects induced in rat calvaria.

In addition, absorbable polymers for bone tissue engineering must ensure mechanical stability while degrading, thus keeping defect site stability during bone regeneration [49, 50]. We examined here which 3D-PLA printed porous scaffold designs provided the best mechanical stability over time during their degradation.

Early investigations on 3D-PLA scaffolds suggested an ideal pore size in the 100-μm range, based on cell size. Subsequent studies suggested that larger pores (100–350 μm) improve cell migration and blood vessel formation [51, 52]. SEM examination revealed that the 3D PLA printed scaffolds showed larger filaments and reduced pore size with respect to the features of the PLA polymer during extrusion and subsequent cooling. As a consequence, the scaffolds produced in this study should show adequate cellular migration and provide nutrients after in vivo implantation [53].

Since the influence of nanoscale topography is crucial for bone substitute biomaterials, its role on our 3D-PLA scaffolds must be considered [54]. The scaffold degradation was revealed by mass loss and decrease of extract pH, and tested at increasing times over a 122-days period. At the end of the study, the mass was significantly reduced in all groups. The pH reduction confirmed the lactide loss [55].

Noteworthy, the degradation did not have a major impact on the mechanical properties over the 112-days time frame.

Scaffold-host tissue compatibility is a fundamental characteristic. Therefore, when using a degradable material its degradation byproducts must not induce a cytotoxic response. This work evaluated the potential cytotoxicity elicited during the absorption of PLA. Lactic acid is one of the PLA hydrolytical degradation byproducts [56]. This is a key point, as one indication of degradation of polyesters is a decrease in pH as seen in this study. The reduced cell metabolic activity after incubation with the eluates was statistically lower than control cells; however, it must be considered that this result was not influenced by the increasing degradation time for scaffold design C. The slow absorption of the tested PLA allowed for mechanical stability over the estimated bone formation and maturation time. Moreover, this pattern reduced the cytotoxicity potential due to slow release of acid. The slow absorption rate and host metabolization of lactic acid are fundamental for a low cytotoxic response in vivo. For PLA in vivo, the acidic byproduct is lactic acid and it would be metabolized through the Cori cycle; this process cannot be replicated in vitro [57]. Eventually, our study demonstrated that the increase of PLA byproducts over time did not induce an increased cytotoxic response for design C and SEM evaluation of 3D-PLA + hGMSCs constructs demonstrated good anchorage, morphological characteristics, and bridging of the cultured cells. Moreover, the biochemical analysis showed an increased expression of RUNX2 in design C.

MSCs have been used for autologous therapy in combination with platelet-rich plasma and/or scaffolds in distraction osteogenesis [58, 59]. Among the various tissues from which MSCs can be isolated, growing attention has been paid to dental tissues including periodontal ligament, dental pulp, and gingiva owing to the minimally invasive procedure involved in their collection, and remarkable differentiation ability towards neurogenic and other cells [60]. Recently, animal data confirm that periodontal ligament stem cells seeded onto 3D scaffolds in the mouse calvaria undergo precocious osteointegration and vascularization [36, 61]. This effect may be due to the ability of the stem cells to undergo osteogenic differentiation, but also to their immunomodulatory and anti-inflammatory properties.

In particular, several findings suggest that, in general, MSCs exert their action mainly through paracrine signaling by EVs and their soluble secretory products, also called a ‘secretome’, containing a pool of soluble cytokines [41]. Released membrane vesicles from eukaryotic cells, as exosomes, microparticles, microvesicles, and apoptotic bodies, can be retained as a dynamic extracellular vesicular compartment, strategic for their paracrine or autocrine biological effects in tissue metabolism [62]. EVs are characterized by the presence of specific membrane-associated proteins, such as CD9, CD63, CD81, and tumor suppressor gene 101 (TSG101) [63].

In vitro, mineralizing osteoblast exosomes are capable of entering into bone marrow stromal cells to induce them to the osteoblast phenotype, with trough upregulation of b-catenin in recipient cells representing a potential therapeutic approach [64]. Recently, it has also been demonstrated that the beta tricalcium phosphate (β-TCP) scaffold functionalized with human-induced pluripotent stem cell-derived MSCs promotes bone repair and regeneration in a rat model of calvarial bone defects [64]. The underlying mechanism through which exosomes enhance the osteoinductive activity of β-TCP and promote bone regeneration could be due to the activation of endogenous bone marrow MSCs in the bone defect site. This was suggested by the observation that exosomes could be released from the exosome/β-TCP complex and then be internalized by bone marrow MSCs [65].

To improve the internalization and performance of the EVs we have complexed them with PEI. As far as PEI-engineered EVs are concerned, many factors such as molecular weight, degree of branching, zeta potential, particle size, cationic charge density, molecular structure, sequence, and conformational flexibility have all been shown to affect PEI efficiency and cytotoxicity when used as a transfecting agent [66]. We found that the best compromise between effectiveness and toxicity was a concentration of 0.05 mg/mL PEI that conferred to EVs a slight increase in zeta-potential from −10.7 ± 0.9 mV to −1.2 ± 0.9 mV with a tolerable viability of the recipient cells (5–10%). The improved internalization of PEI-EVs with respect to EVs may be ascribed to the capacity of PEI, being a cationic species, to favor internalization via proteoglycan binding [67]. The subsequent internalization mechanisms proposed for EVs are attachment or fusion with the target cell membrane, delivering exosomal proteins to the recipient cell [68, 69], or internalization by the recipient cells by mechanisms such as endocytosis [70]. In the present study, labeled PEI-EVs were demonstrated to be internalized mainly by endocytosis, being highly represented after incubation in the recipient cells. The “proton sponge” effect that characterize PEIs is then essential for the endosomal content escape ability and could be related to the better efficiency of PEI-EVs as compared with nonfunctionalized EVs [71].

Our report based on in vitro and in vivo functional studies focuses on the behavior of the living construct constituted from 3D-PLA + hGMSCs and engineered or nonengineered EVs. Alizarin Red S staining showed that after 6 weeks of culture calcium depositions are more evident in the presence of 3D-PLA and EVs or PEI-EVs. In fact, mounting evidence suggests that EVs alone are capable of promoting proliferation and migration of cells as well as osteogenesis and angiogenesis [72]. The transcriptome profile of genes involved in osteoblast differentiation and ossification in a 3D-PLA living construct enriched with EVs and PEI-EVs was examined by the NGS platform. Our results showed that 31 genes were differentially expressed between the examined groups. Specifically, the GO analysis demonstrated that biomaterial enriched with PEI-EVs + hGMSCs induced upregulation of all 31 identified genes involved in the regulation of ossification as well as in the ossification processes (FHL2, BMP2, TWSG1, CCDC47, FAM20C, ERCC2, LEP, TOB2, IMPAD1, CHRDL1, MINPP1, HIRA, MYBBP1A, JAG1, MEF2C, SUCO, SFRP1, SOX9, SIX2, RHOA, PDLIM7, IFT80, SMAD1, HDAC7, ASF1A, ID3, SNAI1, PEX7, RPL38, BMP2K, and BCAP29) when compared with hGMSCs.

Likewise, our results showed that, among the examined 31 genes, 19 genes involved in the “regulation of osteoblast differentiation” and “osteoblast differentiation” (FHL2, BMP2, TWSG1, CCDC47, FAM20C, HIRA, MYBBP1A, JAG1, MEF2C, SUCO, SFRP1, PDLIM7, SMAD1, IFT80, HDAC7, ASF1A, ID3, SNAI1, and BCAP29) were upregulated by the biomaterial in PEI-EVs + hGMSCs compared with hGMSCs.

Moreover, we found that 9 genes that were reported to be upregulated during osteogenesis and some genes reported to be involved in osteoblast differentiation through TGF-β signaling were expressed at higher levels in 3D-PLA + PEI-EVs + hGMSCs compared with hGMSCs [47, 73]. In addition, we evaluated the gene expression profiles of ECM and adhesion molecules in the 3D-PLA + PEI-EVs + hGMSCs group compared with control cells. A total of 20 differentially expressed genes were found in the 3D-PLA + PEI-EVs + hGMSCs group. Upregulated genes were those that encode for integrin (ITGA6), basement membrane laminins (LAMB3, LAMA1, and LAMC1), membrane proteins that mediate cell-to-cell and cell-to-matrix interactions (CTNNA1, VCAN, CD44, and THBS2), and inhibitor of the matrix metalloproteinases (TIMP3). Downregulated genes were those that encode for cell-cell adhesion (ITGA3, ITGB5, ITGAV, ACTB, CTNNB1, and CTGF) and for the ECM constituents of basement membrane (LAMA3, TNC, GAPDH, and COL4A2). All these results suggest the prospective role of 3D-PLA biomaterial enriched with PEI-EVs + hGMSCs in inducing the regulation of adhesion molecules, ECM, and osteogenic genes.

In light of the above findings, we investigated the osteogenic effects of 3D-PLA scaffold infiltrated or not with hGMSCs-derived extracellular vesicles. In vivo studies testing biomaterials in the complex environment of the body are considered strategic to understanding the biological machinery involved in bone regeneration. Previously, we have demonstrated that periodontal ligament stem cells implanted in the mouse do not show immunogenic effects, and after 3 weeks a massive number of cells with different sizes and features and maturation degrees were detected in the mouse calvaria implanted with hPDLSCs/DB [36]. Here, we demonstrated that hGMSCs seeded onto 3D-PLA can induce a regenerative bone process but the presence of EVs and, principally, PEI-EVs also activates local bone induction significantly contributing to the regeneration process. In fact, the 3D-PLA scaffold implanted in the mouse calvaria does not show immunogenic effects and, after 6 weeks, numerous cells secrete ECM that initiates osteogenic mineralization. In addition, the presence of the EVs and, more specifically, PEI-EVs linked to the 3D-PLA scaffold improve the mineralization process as well as the development of an extensive vascular network that is indicative of osteointegration, as recently shown by Xie et al. [28]. The key role of PEI-EVs in bone repair was also shown by CT analysis, with a complete repair seen in both the 3D-PLA + PEI-EVs and 3D-PLA + PEI-EVs + hGMSCs groups. CT analysis showed the presence of damage in the 3D-PLA + EVs group, but in the 3D-PLA + EVs + hGMSCs group the bone regeneration was similar to the groups with PEI-EVs. These findings suggest that the EVs fraction can contribute to osteogenic regeneration and, in particular, that the PEI-engineered EVs are responsible for a more rapid evolution and a major degree of maturation of new bone tissue. In fact, after 6 weeks of in vivo implantation, formation of new bone nodules and blood vessels were evident in calvariae samples grafted with 3D-PLA + PEI-EVs. Our data are therefore in agreement with the promising results on the pro-osteogenic impact of EVs visualized in animal models; EVs stimulate bone regeneration whereas PEI-EVs induce the bone apposition and emphasize the proangiogenic capacity, thus showing that scaffolds coated with PEI-EVs could represent a new tool in critical-size bone defects [61]. Similarly, PEI-decorated graphene oxide is a potent inducer of stem cell osteogenesis leading to nearly double the alkaline phosphatase expression and mineralization [74]. Along these lines, it could be concluded that PEI-EVs play a critical role in cell fate determination; they are not inert [75], but rather they enrich the chemical and physical properties of our novel 3D-PLA printed porous scaffolds.

## Conclusions

With the limitations of this study, we have shown that 3D-PLA printed porous scaffolds and the beneficial effect of EVs and PEI-EVs on osteogenic commitment could represent a novel platform in the study of personalized stem cell-free therapy in bone tissue regeneration.

## Additional files

Additional file 1: Cytotoxicity of degradation byproducts. (A–E) Graphs reporting the metabolic activity of cells exposed to the extract of degrading PLA scaffolds at different endpoint for each design. (F) MTT assay performed on hGMSCs directly exposed to the 3D PLA scaffold at different endpoint. (JPEG 1168 kb)

Additional file 2: 3D PLA and hGMSCs interactions. SEM micrographs at low (A) and high magnifications (B) showing cell adhesion on the scaffold surface. Scaffold surface without hGMSCs are reported in the inset in section A. A, magnification 300×; B, magnification 750×. Scale bar = 10 μm. (JPEG 1188 kb)

Additional file 3: Gene expression. (A) The expression value of genes for “regulation of ossification” and “ossification” differentially expressed between 3D-PLA+ EVs + hGMSCs and 3D-PLA+ PEI-EVs + hGMSCs and compared with hGMSCs. (B) The expression value of genes for “regulation of osteoblast differentiation” and “osteoblast differentiation” differentially expressed between 3D-PLA+ EVs + hGMSCs and 3D-PLA+ PEI-EVs + hGMSCs and compared with hGMSCs. (JPEG 769 kb)

Additional file 4: **Table S1.** The differential gene expression between 3D-PLA+ EVs + hGMSCs and 3D-PLA+ PEI-EVs + hGMSCs compared with hGMSCs is given as expression value and fold change expressed in logarithm with base 2 (FC Log2). Gene ontology (GO) processes indicate the gene classification in the “regulation of ossification” and “ossification”. Instead, the statistical significance is indicated by the false discovery rate (FDR), *q* values ≤ 0.05 were considered statistically significant. **Table S2.** The differential gene expression between 3D-PLA+ EVs + hGMSCs and 3D-PLA+ PEI-EVs + hGMSCs compared with hGMSCs is given as expression value and fold change expressed in logarithm with base 2 (FC Log2). Gene ontology (GO) processes indicate the gene classification in the “regulation of osteoblast differentiation” and “osteoblast differentiation”. Instead, the statistical significance is indicated by the false discovery rate (FDR), *q* values ≤ 0.05 were considered statistically significant. **Table S3.** The differential gene expression between 3D-PLA+ EVs + hGMSCs and 3D-PLA+ PEI-EVs + hGMSCs compared with hGMSCs is given as expression value and fold change expressed in logarithm with base 2 (FC Log2). The statistical significance is indicated by the false discovery rate (FDR), *q* values ≤ 0.05 were considered statistically significant. **Table S4.** The differential gene expression in 3D-PLA+ PEI-EVs + hGMSCs compared with hGMSCs is given in fold change expressed in logarithm with base 2 (FC Log2). The statistical significance is indicated by the false discovery rate (FDR), *q* values ≤ 0.05 were considered statistically significant. (DOCX 59 kb)

Additional file 5: Gene expression. Expression value of genes activated during osteogenesis and osteoblast differentiation in 3D-PLA+ EVs + hGMSCs and 3D-PLA+ PEI-EVs + hGMSCs and compared with hGMSCs (*q* ≤ 0.05, Benjamini–Hochberg false discovery rate). (JPEG 319 kb)

Additional file 6: Gene analysis. Differential regulation of genes coding for adhesion molecules and ECM proteins in the 3D-PLA + PEI-EVs + hGMSCs group compared with hGMSCs cells. Upregulated transcripts shown in red, downregulated transcripts shown in green (*q* ≤ 0.05, Benjamini–Hochberg false discovery rate). (JPEG 271 kb)

## Abbreviations

3D: Three-dimensional
AFM: Atomic force microscopy
CLSM: Confocal laser scanning microscopy
CM: Conditioned medium
DLS: Dynamic laser light scattering
ECM: Extracellular matrix
EVs: Extracellular vesicles
GO: Gene ontology
hGMSCs: Human gingival mesenchymal stem cells
mCT: Microcomputed tomography
MSCGM-CD: Mesenchymal stem cell growth medium—chemically defined
MSC: Mesenchymal stem cell
NGS: Next-generation sequencing
PBS: Phosphate-buffered solution
PEI: Polyethyleneimine
PEI-EVs: Polyethyleneimine-engineered extracellular vesicles
PLA: Poly(lactide)
SEM: Scanning electron microscopy
TCP: Tricalcium phosphate
