# Supplementary figures and images for: Three-dimensional printed PLA scaffold and human gingival stem cell-derived extracellular vesicles: a new tool for bone defect repair
Source: Stem Cell Res Ther. 2018 Apr 13;9:104. doi: 10.1186/s13287-018-0850-0 (PMC5899396; doi:10.1186/s13287-018-0850-0)

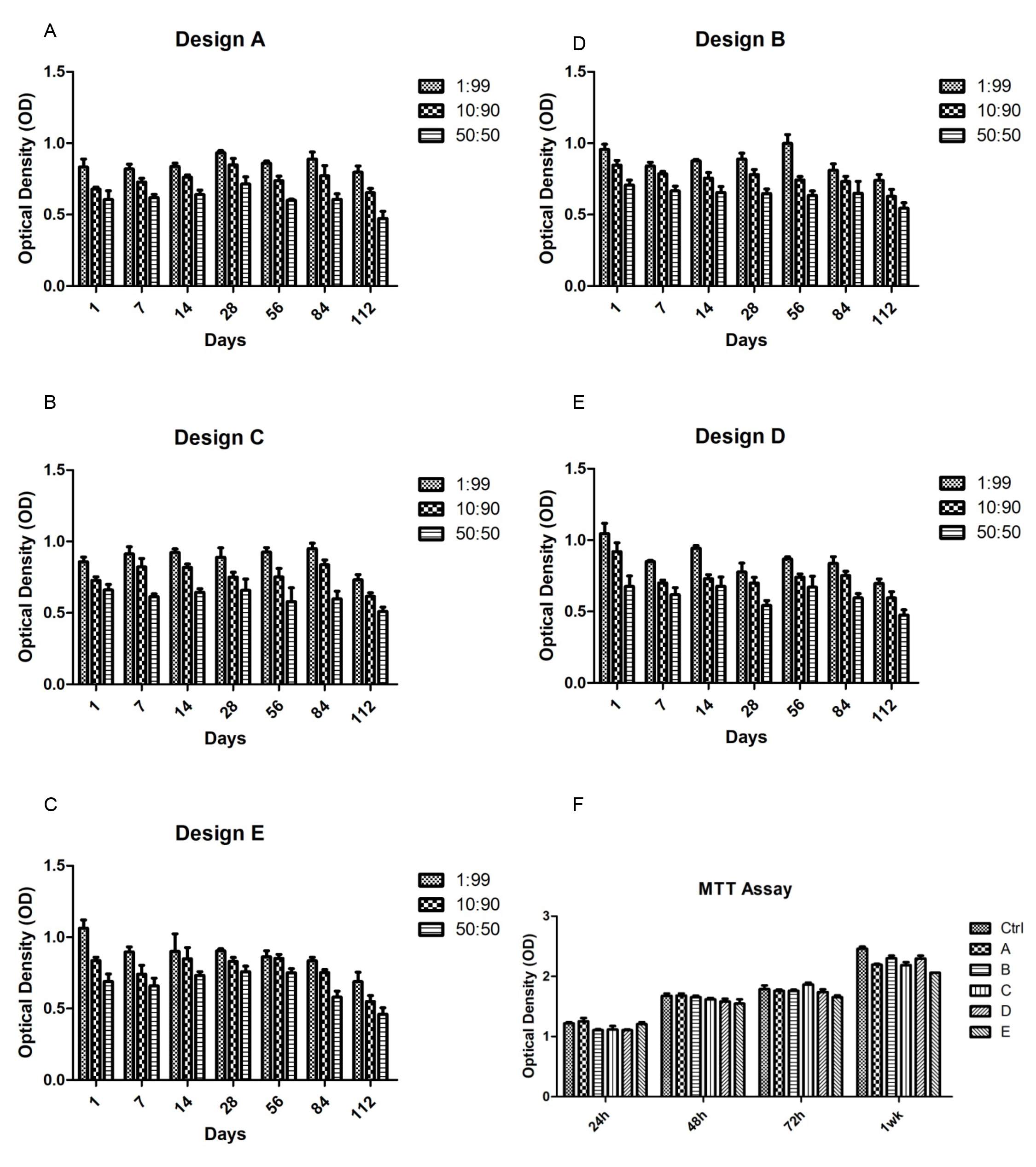

Supplement: Supplementary file 1 — Cytotoxicity of degradation byproducts. (A–E) Graphs reporting the metabolic activity of cells exposed to the extract of degrading PLA scaffolds at different endpoint for each design. (F) MTT assay performed on hGMSCs directly exposed to the 3D PLA scaffold at different endpoint. (JPEG 1168 kb) [file 13287_2018_850_MOESM1_ESM.jpg]

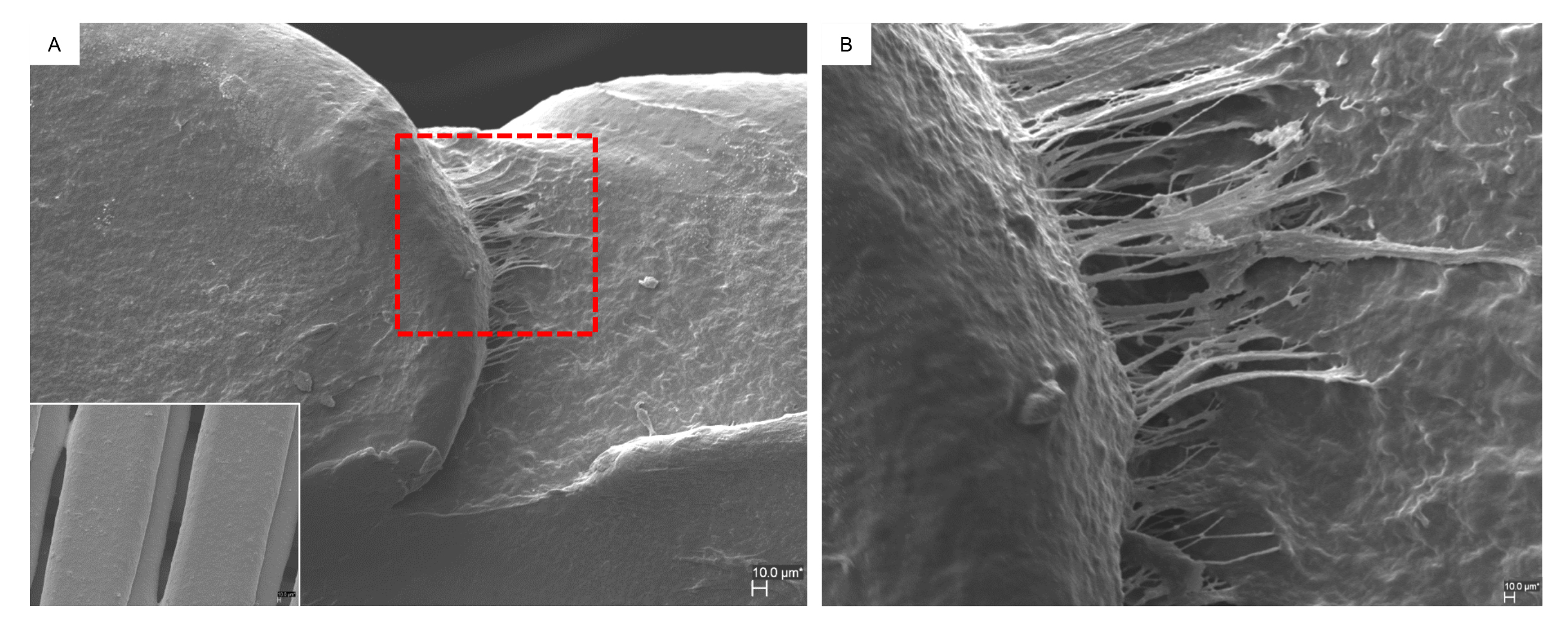

Supplement: Supplementary file 2 — 3D PLA and hGMSCs interactions. SEM micrographs at low (A) and high magnifications (B) showing cell adhesion on the scaffold surface. Scaffold surface without hGMSCs are reported in the inset in section A. A, magnification 300×; B, magnification 750×. Scale bar = 10 μm. (JPEG 1188 kb) [file 13287_2018_850_MOESM2_ESM.jpg]

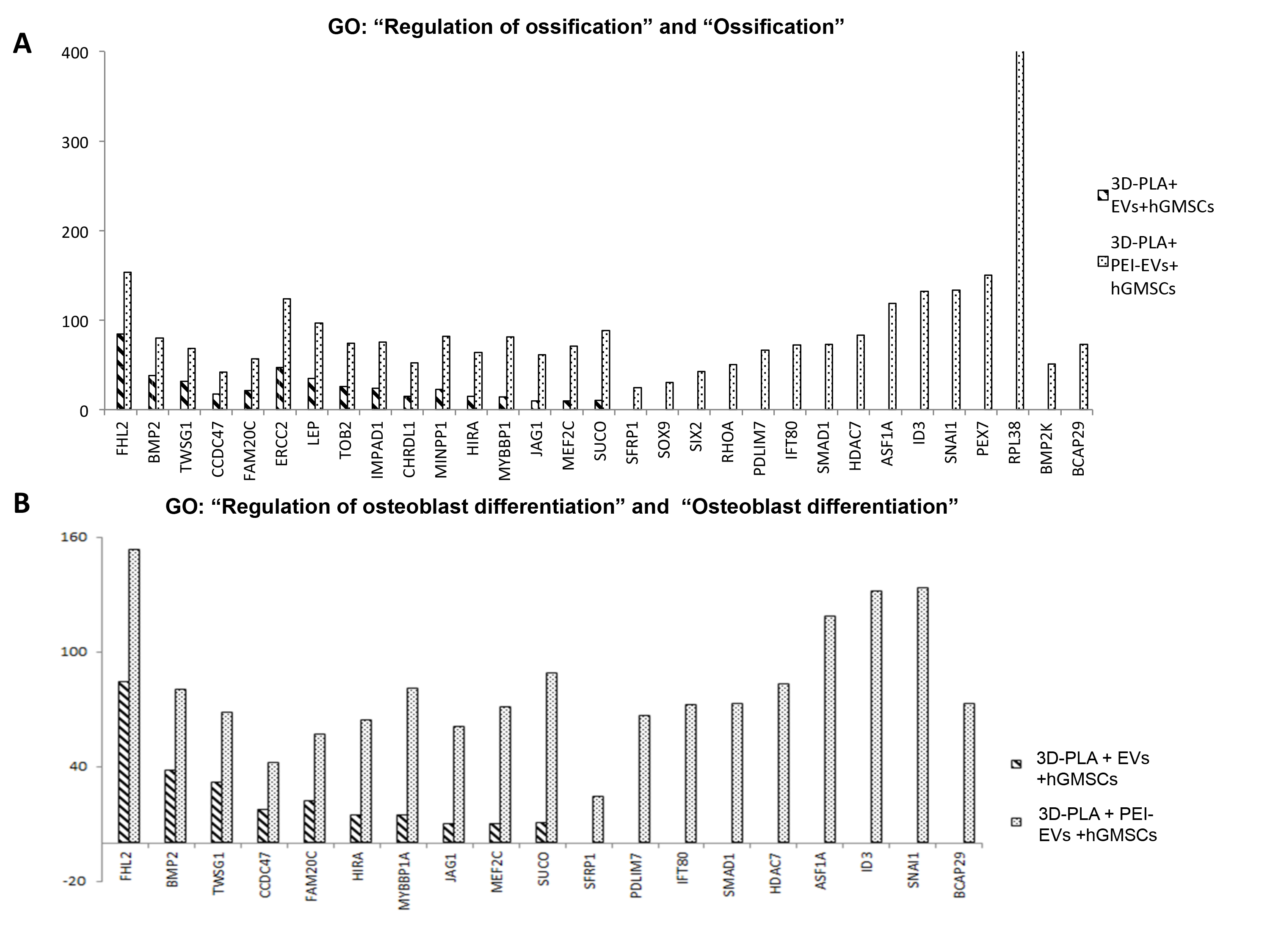

Supplement: Supplementary file 3 — Gene expression. (A) The expression value of genes for “regulation of ossification” and “ossification” differentially expressed between 3D-PLA+ EVs + hGMSCs and 3D-PLA+ PEI-EVs + hGMSCs and compared with hGMSCs. (B) The expression value of genes for “regulation of osteoblast differentiation” and “osteoblast differentiation” differentially expressed between 3D-PLA+ EVs + hGMSCs and 3D-PLA+ PEI-EVs + hGMSCs and compared with hGMSCs. (JPEG 769 kb) [file 13287_2018_850_MOESM3_ESM.jpg]

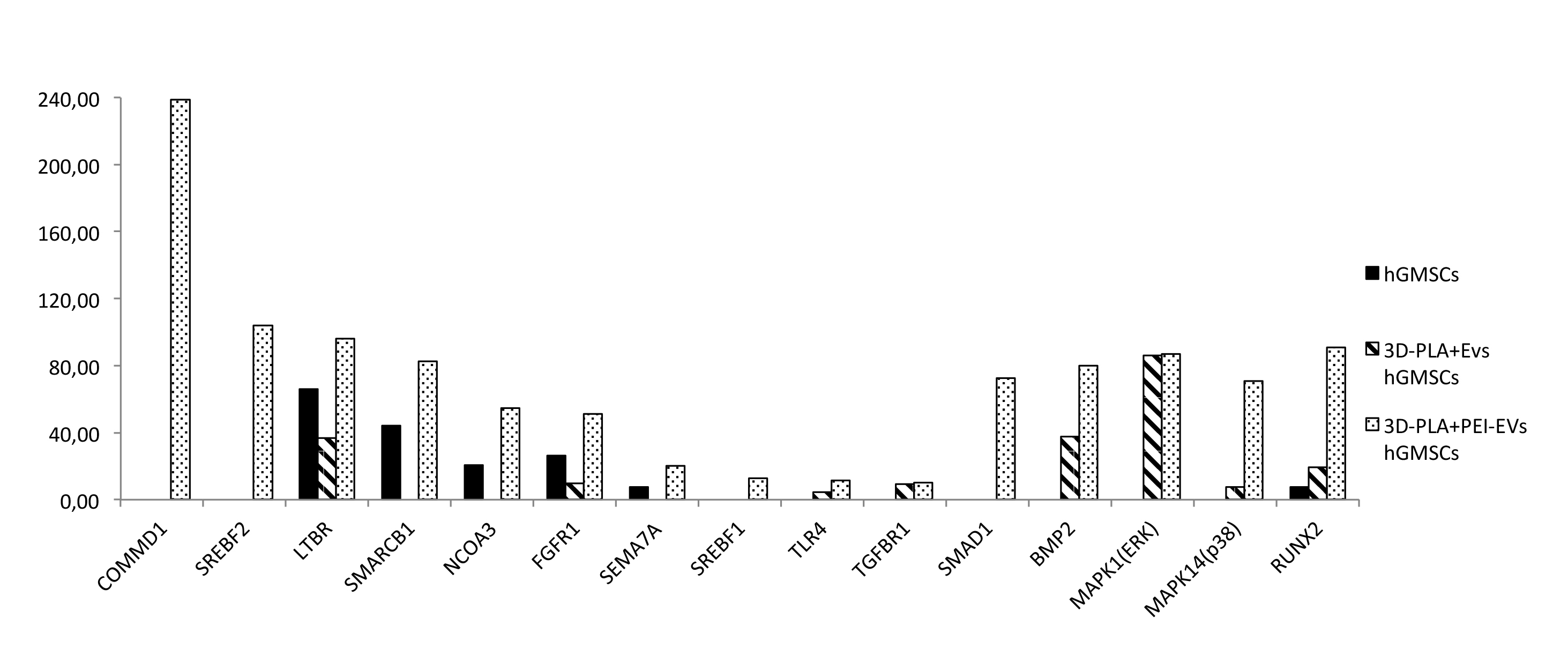

Supplement: Supplementary file 5 — Gene expression. Expression value of genes activated during osteogenesis and osteoblast differentiation in 3D-PLA+ EVs + hGMSCs and 3D-PLA+ PEI-EVs + hGMSCs and compared with hGMSCs (q ≤ 0.05, Benjamini–Hochberg false discovery rate). (JPEG 319 kb) [file 13287_2018_850_MOESM5_ESM.jpg]

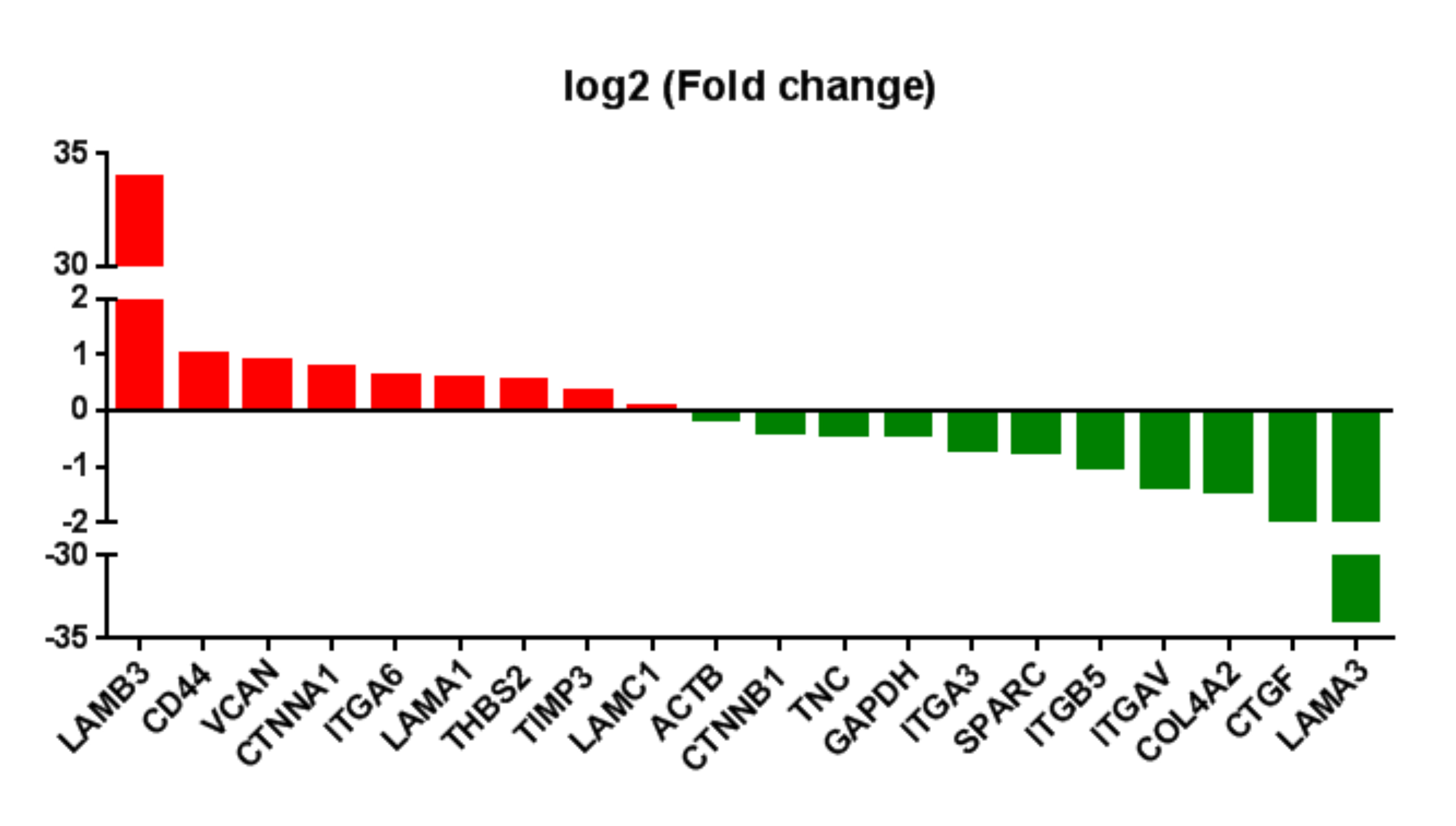

Supplement: Supplementary file 6 — Gene analysis. Differential regulation of genes coding for adhesion molecules and ECM proteins in the 3D-PLA + PEI-EVs + hGMSCs group compared with hGMSCs cells. Upregulated transcripts shown in red, downregulated transcripts shown in green (q ≤ 0.05, Benjamini–Hochberg false discovery rate). (JPEG 271 kb) [file 13287_2018_850_MOESM6_ESM.jpg]
