# Supplementary material for: Three-dimensional printed PLA scaffold and human gingival stem cell-derived extracellular vesicles: a new tool for bone defect repair
Source: Stem Cell Res Ther. 2018 Apr 13;9:104. doi: 10.1186/s13287-018-0850-0 (PMC5899396; doi:10.1186/s13287-018-0850-0)
Supplement: Supplementary file 4 — Table S1. The differential gene expression between 3D-PLA+ EVs + hGMSCs and 3D-PLA+ PEI-EVs + hGMSCs compared with hGMSCs is given as expression value and fold change expressed in logarithm with base 2 (FC Log2). Gene ontology (GO) processes indicate the gene classification in the “regulation of ossification” and “ossification”. Instead, the statistical significance is indicated by the false discovery rate (FDR), q values ≤ 0.05 were considered statistically significant. Table S2. The differential gene expression between 3D-PLA+ EVs + hGMSCs and 3D-PLA+ PEI-EVs + hGMSCs compared with hGMSCs is given as expression value and fold change expressed in logarithm with base 2 (FC Log2). Gene ontology (GO) processes indicate the gene classification in the “regulation of osteoblast differentiation” and “osteoblast differentiation”. Instead, the statistical significance is indicated by the false discovery rate (FDR), q values ≤ 0.05 were considered statistically significant. Table S3. The differential gene expression between 3D-PLA+ EVs + hGMSCs and 3D-PLA+ PEI-EVs + hGMSCs compared with hGMSCs is given as expression value and fold change expressed in logarithm with base 2 (FC Log2). The statistical significance is indicated by the false discovery rate (FDR), q values ≤ 0.05 were considered statistically significant. Table S4. The differential gene expression in 3D-PLA+ PEI-EVs + hGMSCs compared with hGMSCs is given in fold change expressed in logarithm with base 2 (FC Log2). The statistical significance is indicated by the false discovery rate (FDR), q values ≤ 0.05 were considered statistically significant. (DOCX 59 kb) [file 13287_2018_850_MOESM4_ESM.docx]

| **Property** | **Test Standard** | **Unit** | **Values (50%RH)** |
| --- | --- | --- | --- |
| Tensile Strength | ISO 527 | MPa | 52 |
| Elongation Strength | ISO 527 | % | >20 |
| Flexural Stress | ISO 178 | MPa | 85 |
| IZOD impact, notched | ISO 180/1A | kJ/m^2^ | 22 |
| H.D.T. Method A (1.8 MPa) | ISO 75 | °C | 65 |
| Density | ISO 1183 | g/cm^3^ | 1,25 |
| Fire Resistance (3.2mm) | UL 94 | - | HB |
| Melt Temperature Range | - | °C | 220-230 |

**Table 1.** Physical characteristics of the investigated poly-(lactide)

| **Sample** | **% New Bone** | **% 3D-PLA+ECM** |
| --- | --- | --- |
| 3D-PLA | 0 | 100 |
| 3D-PLA/hGMSCs | 3,07519116 | 96,9248088 |
| 3D-PLA/Evs | 0,81936967 | 99,1806303 |
| 3D-PLA/EVs/hGMSCs | 1,73274672 | 98,2672533 |
| 3D-PLA/PEI-EVs | 12,2733915 | 87,7266085 |
| 3D-PLA/PEI-EVs/hGMSCs | 9,71949016 | 90,2805098 |

**Table 2**. Histomorphometric analysis.

| **Gene ID**  **(Entrez)** | **Name** | **Description** | **GO Processes** | **Gene expression value**  **hGMSCs** | **Gene expression value**  **3D-PLA+ EVs + hGMSCs** | **Gene expression value**  **3D-PLA+ PEI-EVs + hGMSCs** | **Log_2_ (fold change)**  **hGMSCs vs 3D-PLA+ EVs + hGMSCs** | **Log_2_ (fold change)**  **hGMSCs vs 3D-PLA+ PEI-EVs + hGMSCs** | **FDR**  **hGMSCs vs 3D-PLA+ EVs + hGMSCs** | **FDR**  **hGMSCs vs 3D-PLA+ PEI-EVs+ hGMSCs** |
| --- | --- | --- | --- | --- | --- | --- | --- | --- | --- | --- |
| 2274 | FHL2 | four and a half LIM domains 2 | ossification | 0,00 | 84,41 | 153,21 | 36,30 | 37,16 | 1,24E-04 | 0,027652 |
| 650 | BMP2 | bone morphogenetic protein 2 | regulation of ossification and ossification | 0,00 | 37,77 | 80,00 | 35,14 | 36,22 | 1,24E-04 | 0,043802 |
| 57045 | TWSG1 | twisted gastrulation BMP signaling modulator 1 | regulation of ossification and ossification | 0,00 | 31,33 | 68,16 | 34,86 | 35,98 | 1,71E-03 | 0,01127 |
| 57003 | CCDC47 | coiled-coil domain containing 47 | ossification | 0,00 | 17,30 | 42,00 | 34,01 | 35,29 | 2,07E-03 | 0,000238 |
| 56975 | FAM20C | golgi associated secretory pathway kinase | regulation of ossification | 0,00 | 21,59 | 56,66 | 34,33 | 35,72 | 2,38E-04 | 0,000124 |
| 2068 | ERCC2 | ERCC excision repair 2, TFIIH core complex helicase subunit | ossification | 0,00 | 47,06 | 123,56 | 35,45 | 36,85 | 1,10E-02 | 0,000124 |
| 3952 | LEP | leptin | ossification | 0,00 | 34,52 | 97,00 | 35,01 | 36,50 | 1,24E-04 | 0,02897 |
| 10766 | TOB2 | transducer of ERBB2, 2 | regulation of ossification | 0,00 | 25,52 | 73,96 | 34,57 | 36,10 | 1,24E-04 | 0,000124 |
| 54928 | IMPAD1 | inositol monophosphatase domain containing 1 | ossification | 0,00 | 23,76 | 75,68 | 34,47 | 36,14 | 1,06E-02 | 0,017333 |
| 91851 | CHRDL1 | chordin like 1 | ossification | 0,00 | 14,68 | 52,00 | 33,77 | 35,60 | 1,89E-03 | 0,032525 |
| 9562 | MINPP1 | multiple inositol-polyphosphate phosphatase 1 | ossification | 0,00 | 22,57 | 82,00 | 34,39 | 36,25 | 8,17E-03 | 0,032395 |
| 7290 | HIRA | histone cell cycle regulator | ossification | 0,00 | 14,61 | 64,00 | 33,77 | 35,90 | 1,24E-04 | 0,001055 |
| 10514 | MYBBP1A | MYB binding protein 1a | ossification | 0,00 | 14,25 | 81,00 | 33,73 | 36,23 | 6,88E-03 | 0,005678 |
| 182 | JAG1 | jagged 1 | regulation of ossification | 0,00 | 9,62 | 61,00 | 33,16 | 35,83 | 2,46E-02 | 0,00076 |
| 4208 | MEF2C | myocyte enhancer factor 2C | regulation of ossification and ossification | 0,00 | 10,01 | 71,12 | 33,22 | 36,04 | 1,24E-03 | 0,001619 |
| 51430 | SUCO | SUN domain containing ossification factor | regulation of ossification and ossification | 0,00 | 10,49 | 88,58 | 33,29 | 36,37 | 5,57E-04 | 0,004757 |
| 6422 | SFRP1 | secreted frizzled related protein 1 | ossification, | 0,00 | 0,00 | 24,30 | 0 | 34,50 | 3,06E-03 | 0,025252 |
| 6662 | SOX9 | SRY-box 9 | ossification | 0,00 | 0,00 | 30,34 | 0 | 34,82 | 1,24E-04 | 0,011423 |
| 10736 | SIX2 | SIX homeobox 2 | regulation of ossification | 0,00 | 0,00 | 42,25 | 0 | 35,29 | 4,53E-04 | 0,016284 |
| 387 | RHOA | ras homolog family member A | ossification | 0,00 | 0,00 | 50,48 | 0 | 35,55 | 1,24E-04 | 0,00076 |
| 9260 | PDLIM7 | PDZ and LIM domain 7 | regulation of ossification and ossification | 0,00 | 0,00 | 66,41 | 0 | 35,95 | 3,69E-02 | 0,011637 |
| 57560 | IFT80 | intraflagellar transport 80 | ossification | 0,00 | 0,00 | 72,41 | 0 | 36,08 | 1,24E-04 | 0,01659 |
| 4086 | SMAD1 | SMAD family member 1 | regulation of ossification and ossification | 0,00 | 0,00 | 72,60 | 0 | 36,07 | 1,24E-04 | 0,013474 |
| 51564 | HDAC7 | histone deacetylase 7 | regulation of ossification | 0,00 | 0,00 | 83,34 | 0 | 36,28 | 1,24E-04 | 0,010254 |
| 25842 | ASF1A | anti-silencing function 1A histone chaperone | ossification | 0,00 | 0,00 | 118,45 | 0 | 36,79 | 1,24E-04 | 0,032525 |
| 3399 | ID3 | inhibitor of DNA binding 3, HLH protein | regulation of ossification | 0,00 | 0,00 | 131,82 | 0 | 36,94 | 1,24E-04 | 0,015688 |
| 6615 | SNAI1 | snail family transcriptional repressor 1 | ossification | 0,00 | 0,00 | 133,55 | 0 | 36,95 | 1,15E-03 | 0,014274 |
| 5191 | PEX7 | peroxisomal biogenesis factor 7 | ossification | 0,00 | 0,00 | 150,30 | 0 | 37,12 | 1,24E-04 | 0,019168 |
| 6169 | RPL38 | ribosomal protein L38 | ossification | 0,00 | 0,00 | 401,99 | 0 | 38,54 | 1,24E-04 | 0,007366 |
| 55589 | BMP2K | BMP2 inducible kinase | regulation of ossification | 0,00 | 0,00 | 50,75 | 0 | 16,05 | 1,24E-04 | 0,03266 |
| 55973 | BCAP29 | B-cell receptor associated protein 29 | ossification | 0,00 | 0,00 | 72,94 | 0 | 13,90 | 7,20E-03 | 0,001708 |

**Additional Table 1**. The differential gene expression between 3D-PLA+ EVs + hGMSCs and 3D-PLA+ PEI-EVs + hGMSCs compared to hGMSCs is given in Expression value and Fold Change expressed in logarithm with base 2 (FC Log2). Gene ontology (GO) processes indicate the gene classification in the “regulation of ossification” and “ossification”. Instead, the statistical significance is indicated by the False Discovery Rate (FDR), q-values ≤ 0.05 were considered statistically significant.

| **Gene ID**  **(Entrez)** | **Name** | **Description** | **GO Processes** | **Gene expression value**  **hGMSCs** | **Gene expression value**  **3D-PLA+ EVs + hGMSCs** | **Gene expression value**  **3D-PLA+ PEI-EVs + hGMSCs** | **Log_2_ (fold change)**  **hGMSCs vs 3D-PLA+ EVs + hGMSCs** | **Log_2_ (fold change)**  **hGMSCs vs 3D-PLA+ PEI-EVs + hGMSCs** | **FDR**  **hGMSCs vs 3D-PLA+ EVs + hGMSCs** | **FDR**  **hGMSCs vs 3D-PLA+ PEI-EVs+ hGMSCs** |
| --- | --- | --- | --- | --- | --- | --- | --- | --- | --- | --- |
| 2274 | FHL2 | four and a half LIM domains 2 | osteoblast differentiation | 0,00 | 84,41 | 153,21 | 36,30 | 37,16 | 1,24E-04 | 0,027652 |
| 650 | BMP2 | bone morphogenetic protein 2 | regulation of osteoblast differentiation and osteoblast differentiation | 0,00 | 37,77 | 80,00 | 35,14 | 36,22 | 1,24E-04 | 0,043802 |
| 57045 | TWSG1 | twisted gastrulation BMP signaling modulator 1 | regulation of osteoblast differentiation | 0,00 | 31,33 | 68,16 | 34,86 | 35,98 | 1,71E-03 | 0,01127 |
| 57003 | CCDC47 | coiled-coil domain containing 47 | osteoblast differentiation | 0,00 | 17,30 | 42,00 | 34,01 | 35,29 | 2,07E-03 | 0,000238 |
| 56975 | FAM20C | golgi associated secretory pathway kinase | regulation of osteoblast differentiation | 0,00 | 21,59 | 56,66 | 34,33 | 35,72 | 2,38E-04 | 0,000124 |
| 7290 | HIRA | histone cell cycle regulator | osteoblast differentiation | 0,00 | 14,61 | 64,00 | 33,77 | 35,90 | 1,24E-04 | 0,001055 |
| 10514 | MYBBP1A | MYB binding protein 1a | osteoblast differentiation | 0,00 | 14,25 | 81,00 | 33,73 | 36,23 | 6,88E-03 | 0,005678 |
| 182 | JAG1 | jagged 1 | regulation of osteoblast differentiation | 0,00 | 9,62 | 61,00 | 33,16 | 35,83 | 2,46E-02 | 0,00076 |
| 4208 | MEF2C | myocyte enhancer factor 2C | regulation of osteoblast differentiation and osteoblast differentiation | 0,00 | 10,01 | 71,12 | 33,22 | 36,04 | 1,24E-03 | 0,001619 |
| 51430 | SUCO | SUN domain containing ossification factor | regulation of osteoblast differentiation | 0,00 | 10,49 | 88,58 | 33,29 | 36,37 | 5,57E-04 | 0,004757 |
| 6422 | SFRP1 | secreted frizzled related protein 1 | regulation of osteoblast differentiation and osteoblast differentiation | 0,00 | 0,00 | 24,30 | 0 | 34,50 | 3,06E-03 | 0,025252 |
| 9260 | PDLIM7 | PDZ and LIM domain 7 | regulation of osteoblast differentiation | 0,00 | 0,00 | 66,41 | 0 | 35,95 | 3,69E-02 | 0,011637 |
| 57560 | IFT80 | intraflagellar transport 80 | osteoblast differentiation | 0,00 | 0,00 | 72,41 | 0 | 36,08 | 1,24E-04 | 0,01659 |
| 4086 | SMAD1 | SMAD family member 1 | regulation of osteoblast differentiation and osteoblast differentiation | 0,00 | 0,00 | 72,60 | 0 | 36,07 | 1,24E-04 | 0,013474 |
| 51564 | HDAC7 | histone deacetylase 7 | regulation of osteoblast differentiation | 0,00 | 0,00 | 83,34 | 0 | 36,28 | 1,24E-04 | 0,010254 |
| 25842 | ASF1A | anti-silencing function 1A histone chaperone | osteoblast differentiation | 0,00 | 0,00 | 118,45 | 0 | 36,79 | 1,24E-04 | 0,032525 |
| 3399 | ID3 | inhibitor of DNA binding 3, HLH protein | regulation of  osteoblast differentiation | 0,00 | 0,00 | 131,82 | 0 | 36,94 | 1,24E-04 | 0,015688 |
| 6615 | SNAI1 | snail family transcriptional repressor 1 | osteoblast differentiation | 0,00 | 0,00 | 133,55 | 0 | 36,95 | 1,15E-03 | 0,014274 |
| 55973 | BCAP29 | B-cell receptor associated protein 29 | osteoblast differentiation | 0,00 | 0,00 | 72,94 | 0 | 13,90 | 7,20E-03 | 0,001708 |

**Additional Table 2**. The differential gene expression between 3D-PLA+ EVs + hGMSCs and 3D-PLA+ PEI-EVs + hGMSCs compared to hGMSCs is given in Expression value and Fold Change expressed in logarithm with base 2 (FC Log2). Gene ontology (GO) processes indicate the gene classification in the “regulation of osteoblast differentiation” and “osteoblast differentiation”. Instead, the statistical significance is indicated by the False Discovery Rate (FDR), q-values ≤ 0.05 were considered statistically significant.

| **Gene ID**  **(Entrez)** | **Name** | **Description** | **Gene expression value hGMSCs** | **Gene expression value 3D-PLA+**  **EVs+**  **hGMSCs** | **Gene expression value 3D-PLA+**  **PEI-EVs+**  **hGMSCs** | **Log_2_ (fold change) hGMSCs vs 3D-PLA+**  **EVs+**  **hGMSCs** | **Log_2_ (fold change) hGMSCs vs 3D-PLA+**  **PEI-EVs+**  **hGMSCs** | **FDR hGMSCs vs 3D-PLA+**  **EVs+**  **hGMSCs** | **FDR hGMSCs vs 3D-PLA+**  **PEI-EVs+**  **hGMSCs** |
| --- | --- | --- | --- | --- | --- | --- | --- | --- | --- |
| 150684 | COMMD1 | copper metabolism domain containing 1 | 0.00 | 0.00 | 238.72 | 0.00 | 27.83 | 4.50E-03 | 1.24E-04 |
| 6721 | SREBF2 | sterol regulatory element binding transcription factor 2 | 0.00 | 0.00 | 104.03 | 0.00 | 26.63 | 3.46E-04 | 1.24E-04 |
| 4055 | LTBR | lymphotoxin beta receptor | 65.82 | 36.57 | 96.15 | -0.85 | 0.55 | 7.60E-04 | 9.33E-03 |
| 6598 | SMARCB1 | SWI/SNF related, matrix associated, actin dependent regulator of chromatin, subfamily b, member 1 | 44.19 | 0.00 | 82.74 | -25.40 | 0.90 | 1.24E-04 | 1.24E-04 |
| 8202 | NCOA3 | nuclear receptor coactivator 3 | 20.59 | 0.00 | 54.85 | -24.30 | 1.41 | 1.24E-04 | 1.17E-02 |
| 2260 | FGFR1 | fibroblast growth factor receptor 1 | 26.36 | 9.92 | 51.34 | -1.41 | 0.96 | 1.24E-04 | 1.24E-04 |
| 8482 | SEMA7A | semaphorin 7A | 7.48 | 0.00 | 20.15 | -22.83 | 1.43 | 3.46E-04 | 1.81E-02 |
| 6720 | SREBF1 | sterol regulatory element binding transcription factor 1 | 0.00 | 0.00 | 12.95 | 0.00 | 23.63 | 1.24E-04 | 1.98E-03 |
| 7099 | TLR4 | toll like receptor 4 | 0.00 | 4.56 | 11.54 | 22.12 | 23.46 | 2.07E-03 | 1.24E-04 |
| 7046 | TGFBR1 | transforming growth factor beta receptor 1 | 0.00 | 9.48 | 10.09 | 33.14 | 33.23 | 1.24E-04 | 1.24E-04 |
| 4086 | SMAD1 | SMAD family member 1 | 0.00 | 0.00 | 72.60 | 0.00 | 36.07 | 1.24E-04 | 0.013474 |
| 650 | BMP2 | bone morphogenetic protein 2 | 0.00 | 37.77 | 80.00 | 35.14 | 36.22 | 1.24E-04 | 0.043802 |
| 5594 | MAPK1 | mitogen-activated protein kinase 1 | 0.00 | 85.98 | 87.00 | 36.32 | 36.34 | 1.24E-04 | 1.24E-04 |
| 1432 | MAPK14 | mitogen-activated protein kinase 14 | 0.00 | 7.48 | 71.00 | 32.80 | 36.05 | 1.24E-04 | 2.34E-03 |
| 860 | RUNX2 | runt related transcription factor 2 | 7.37 | 19.36 | 91.00 | 1.39 | 3.63 | 1.24E-04 | 9.58E-04 |

**Additional Table 3.** The differential gene expression between 3D-PLA+ EVs + hGMSCs and 3D-PLA+ PEI-EVs + hGMSCs compared to hGMSCs is given in Expression value and Fold Change expressed in logarithm with base 2 (FC Log2). The statistical significance is indicated by the False Discovery Rate (FDR), q-values ≤ 0.05 were considered statistically significant

| **Gene ID**  **(Entrez)** | **Name** | **Description** | **Log2 (fold change)** | **FDR** | **Regulation** |
| --- | --- | --- | --- | --- | --- |
| 3914 | LAMB3 | laminin subunit beta 3 | 33,89 | 9,58E-04 | Up |
| 960 | CD44 | CD44 molecule | 1,00 | 1,34E-03 | Up |
| 1462 | VCAN | versican | 0,87 | 1,24E-04 | Up |
| 1495 | CTNNA1 | catenin alpha 1 | 0,78 | 1,24E-04 | Up |
| 3655 | ITGA6 | integrin subunit alpha 6 | 0,59 | 1,24E-04 | Up |
| 284217 | LAMA1 | laminin subunit alpha 1 | 0,58 | 5,57E-04 | Up |
| 7058 | THBS2 | thrombospondin 2 | 0,52 | 8,59E-04 | Up |
| 7078 | TIMP3 | TIMP metallopeptidase inhibitor 3 | 0,32 | 1,62E-03 | Up |
| 3915 | LAMC1 | laminin subunit gamma 1 | 0,07 | 1,24E-04 | Up |
| 60 | ACTB | actin beta | -0,13 | 2,72E-02 | Down |
| 1499 | CTNNB1 | catenin beta 1 | -0,38 | 1,24E-04 | Down |
| 3371 | TNC | tenascin C | -0,42 | 2,38E-04 | Down |
| 2597 | GAPDH | glyceraldehyde-3-phosphate dehydrogenase | -0,42 | 1,24E-04 | Down |
| 3675 | ITGA3 | integrin subunit alpha 3 | -0,69 | 1,24E-04 | Down |
| 6678 | SPARC | secreted protein acidic and cysteine rich | -0,71 | 1,24E-04 | Down |
| 3693 | ITGB5 | integrin subunit beta 5 | -1,00 | 1,24E-04 | Down |
| 3685 | ITGAV | integrin subunit alpha V | -1,33 | 1,24E-04 | Down |
| 1284 | COL4A2 | collagen type IV alpha 2 chain | -1,42 | 1,24E-04 | Down |
| 1490 | CTGF | connective tissue growth factor | -1,96 | 6,60E-04 | Down |
| 3909 | LAMA3 | laminin subunit alpha 3 | -33,85 | 4,07E-02 | Down |

**Additional Table 4**. The differential gene expression in 3D-PLA+ PEI-EVs + hGMSCs compared to hGMSCs is given in Fold Change expressed in logarithm with base 2 (FC Log2). The statistical significance is indicated by the False Discovery Rate (FDR), q-values ≤ 0.05 were considered statistically significant.
